# Persulfate-Promoted Carbamoylation/Cyclization of Alkenes: Synthesis of Amide-Containing Quinazolinones

**DOI:** 10.3390/molecules29050997

**Published:** 2024-02-25

**Authors:** Jia-Jun Tang, Meng-Yang Zhao, Ying-Jun Lin, Li-Hua Yang, Long-Yong Xie

**Affiliations:** College of Chemistry and Bioengineering, Hunan University of Science and Engineering, Yongzhou 425100, China13549692061@163.com (L.-H.Y.)

**Keywords:** radical reaction, cascade cyclization, radical, quinazolinones, metal-free

## Abstract

The incorporation of amide groups into biologically active molecules has been proven to be an efficient strategy for drug design and discovery. In this study, we present a simple and practical method for the synthesis of amide-containing quinazolin-4(3*H*)-ones under transition-metal-free conditions. This is achieved through a carbamoyl-radical-triggered cascade cyclization of N3-alkenyl-tethered quinazolinones. Notably, the carbamoyl radical is generated in situ from the oxidative decarboxylative process of oxamic acids in the presence of (NH_4_)_2_S_2_O_8_.

## 1. Introduction

**Figure 1:**
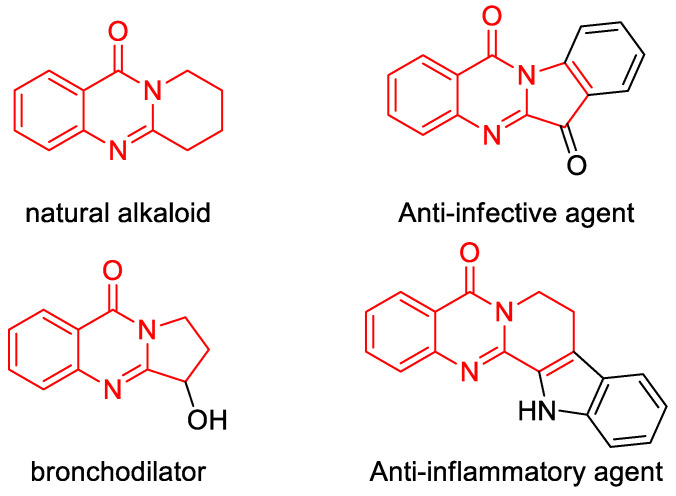
Representative bioactive polycyclic quinoxalin-2(1*H*)-one derivatives.

Quinazolinones, nitrogen-containing heterocycles, are widely present in natural products and pharmaceuticals [1,2,3]. In particular, 2,3-fused quinazolin-4(3*H*)-ones display a wide spectrum of pharmacological and biological properties, including anti-inflammatory, antimicrobial, antiviral, antitumor, and other activities (Figure 1), and have attracted the extensive attention of chemists [4,5,6,7,8].

**Scheme 1:**
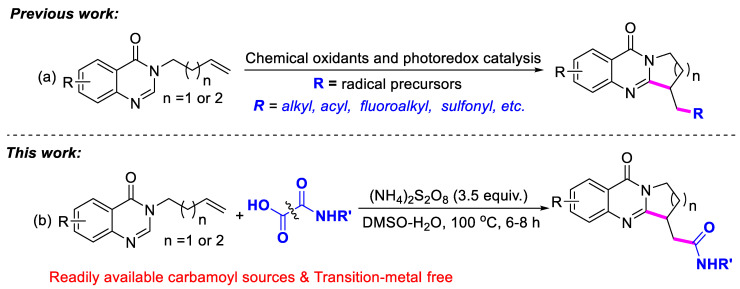
Radical-triggered cascade cyclization of N3-alkenyl-tethered quinazolinones. (**a**) Diverse radical-triggered cascade cyclization of N3-alkenyl-tethered quinazolinones. (**b**) The carbamoyl-radical-triggered cascade cyclization reaction of N3-alkenyl-tethered quinazolinones.

Consequently, remarkable efforts have been devoted to their efficient synthesis in the past few years [9,10,11,12,13,14,15]. Traditionally, 2,3-fused quinazolin-4(3*H*)-ones can be prepared through multiple pathways, such as dehydrogenative cross-coupling reactions [16,17,18,19,20,21], oxidative ring-opening reactions [22,23,24,25,26,27,28], cycloaddition reactions [29,30,31,32], etc. However, these methods suffer from the use of complicated starting materials, harsh reaction conditions, and complex reaction procedures. In recent years, the radical cascade cyclization reaction of N3-alkenyl-tethered quinazolinones has emerged as an attractive strategy for the synthesis of functionalized ring-fused quinazolinone derivatives [33]. In this regard, several important functional groups, including alkyl [34,35,36,37], acyl [38], fluoralkyl [39,40,41,42,43], sulfonyl [44], and sulfonamidation groups [45], were smoothly incorporated into quinazolinone scaffolds (Scheme 1a). Despite these advances, the development of efficient and facile methods for the introduction of some other valuable groups to quinazolinone scaffolds is still in high demand.

On the other hand, amide compounds frequently exist in natural products, pharmaceuticals, and various functional materials [46,47,48]. In contrast to conventional methods for the construction of amide bonds via condensation [49,50] or cross-coupling reaction [51,52,53], which often suffer from the prefunctionalization of substrates and environmentally unfriendly coupling reagents, the radical carbamoylation reaction has been regarded as one of the most efficient approaches to directly introduce an amide group into an organic molecule [54]. In this context, obvious achievements have been made, especially in the carbamoylation of *N*-heterocycles through a Minisci-type reaction process [55,56,57,58]. In recent years, radical difunctionalization reactions of alkenes employing oxamic acids or other carbamoylating reagents such as carbamoyl radical precursors have also provided a promising strategy to introduce amide groups to various complex molecules [59,60]. For instance, in 2021, Wang and co-workers reported persulfate-promoted difunctionalization reactions of ortho-cyanoarylacrylamides with oxamic acids to access a variety of carbamoyl quinoline-2,4-diones [61]. Li and co-workers developed a convenient and practical method for carbamoylated benzimidazo [2,1-a]isoquinolin-6(5*H*)-ones from 2-arylbenzoimidazoles and oxamic acids [62]. Very recently, Anand Singh et al. developed a visible-light-induced cascade carbamoylation/cyclization of acrylamides with 4-carbamoyl-1,4-dihydropyridines as carbamoylation reagents [63]. However, to our knowledge, the carbamoyl-radical-triggered cascade cyclization reaction of N3-alkenyl-tethered quinazolinones to afford amide-containing quinazolinones has never been reported. With our continuing interest in radical chemistry [64,65,66,67,68], herein, we report a metal-free protocol for the synthesis of amide-substituted polycyclic quinazolinones through the cascade radical carbamoylation/cyclization reaction of N3-alkenyl-tethered quinazolinones with oxamic acids as a readily available carbamoyl radical source (Scheme 1b).

## 2. Results and Discussion

Initially, 3-(but-3-en-1-yl)quinazolin-4(3*H*)-one (**1a**) and 2-oxo-2-(phenylamino)acetic acid (**2a**) were selected as the model substrates to screen the optimized reaction conditions, as shown in Table 1. When the reaction was performed at 80 °C under N_2_ atmosphere for 6 h with (NH_4_)_2_S_2_O_8_ as an oxidant in DMSO solvent, the corresponding radical cyclization product **3a** was obtained in 42% isolated yield (entry 1). Then, some other oxidants, including K_2_S_2_O_8_, Na_2_S_2_O_8_, PhI(OAc)_2_, selectfluor reagent, and potassium peroxomonosulfate (Oxone) were investigated, among which K_2_S_2_O_8_ and Na_2_S_2_O_8_ gave low yields in contrast to (NH_4_)_2_S_2_O_8_ (entries 2 and 3 vs. entry 1), and other oxidants failed to give the desired product. Furthermore, various commonly used organic solvents, including CH_3_CN, DCE, DMF, THF, 1,4-dioxane, and NMP were examined. Beyond our expectation, only DMSO was valid for the current transformation and other investigated organic solvents were found unsuitable for this reaction. In addition, water as a solvent also gave 27% yield of **3a** (entry 4). Then, various ratios of DMSO-H_2_O mixed solvents were tested to further enhance the reaction efficiency (entries 5–7). We found an appropriate amount of water is beneficial for the reaction, likely due to the addition of water to DMSO obviously improving the solubility of ammonium persulfate in solvents, while too much water may reduce the solubility of the reactants and result in a worse yield. When the volume ratio of DMSO to H_2_O is 100:1 (*v*/*v*), the yield of **3a** reached a yield of 57% (entry 6). The reaction temperature has a significant influence on the reaction yield. Increasing the reaction temperature to 90 and 100 °C, the yield of **3a** was obtained in 64 and 76% yields, respectively (entries 8 and 9). Further increasing the reaction temperature did not improve the yield (entry 10), while reducing the temperature to 70 °C only gave **3a** in 16% yield (entry 11). We also investigated the amounts of (NH_4_)_2_S_2_O_8_ to the reaction (entries 12–14). We found the yield of **3a** was improved to 82% when the amounts of (NH_4_)_2_S_2_O_8_ was increased to 3.5 equiv. based on **1a** (entry 13). Finally, no reaction occurred in the absence of any oxidant, demonstrating that oxidant was essential for the present reaction (entry 15).

**Scheme 2:**
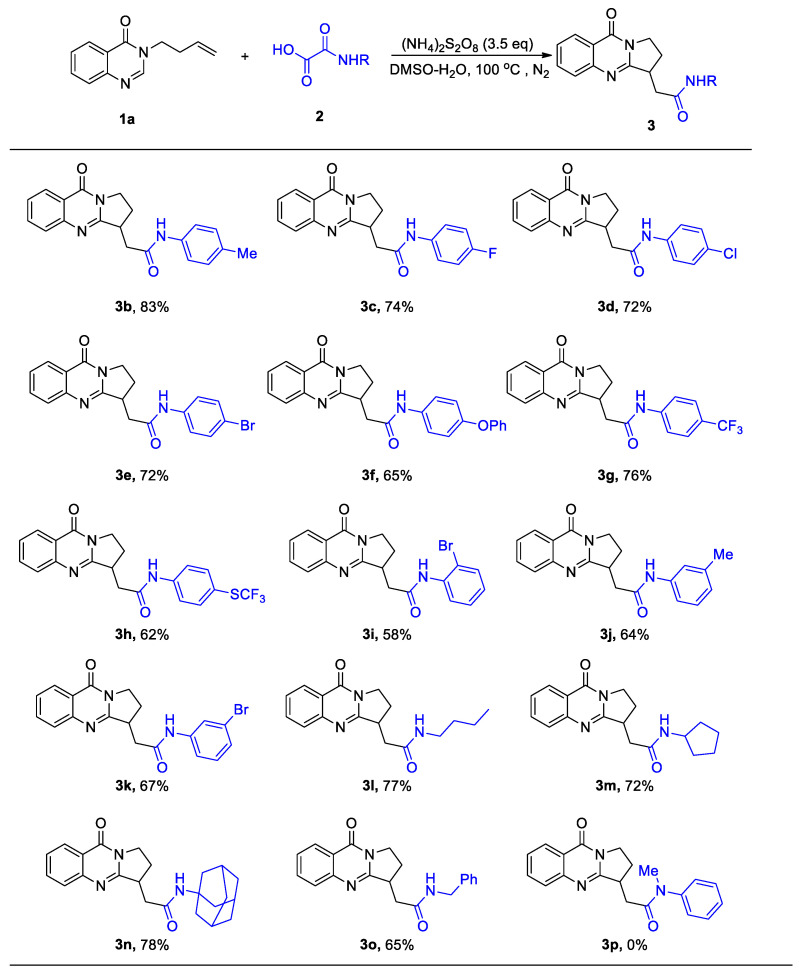
Preparation of **3b**–**3p**. Conditions: **1a** (0.3 mmol, 1 eq), **2** (0.6 mmol, 2 eq), (NH_4_)_2_S_2_O_8_ (1.05 mmol, 3.5 eq), DMSO-H_2_O (3 mL, *v*/*v* 100:1), 100 °C, under N_2_ atmosphere for 6–8 h. Isolated yields were given.

With the optimal reaction conditions in hand (Table 1, entry 22), the substrate scope and limitations of the present reaction were investigated by the reaction of 3-(but-3-en-1-yl)quinazolin-4(3*H*)-one (**1a**) with various oxamic acids. As depicted in Scheme 2, *N*-aryl oxamic acids bearing either electron-donating groups or electron-withdrawing groups at the different positions of aryl rings all reacted well with substrate **1a** to give the corresponding products in 58–83% yields (**3b**–**3k**). Other important functional groups, such as methyl groups (**3b**), halogen atoms (**3c**–**3e**, **3i** and **3k**), trifluoromethyl groups (**3g**), and trifluoromethylthio groups (**3h**), were compatible with the current transformation. In addition, some *N*-alkyl oxamic acids were also found suitable for the reaction and gave the desired products in satisfactory yields (**3l**–**3o**). However, using *N*-disubstituted oxamic acid **2p** as a potential substrate, the expected product was not observed (**3p**). In addition, some *N*-dialkyl oxamic acids obtained from dialkyl amines, such as morpholine, piperidine, and diethylamine, were used for the present reaction, while no reaction occurred to give the corresponding amide products.

**Scheme 3:**
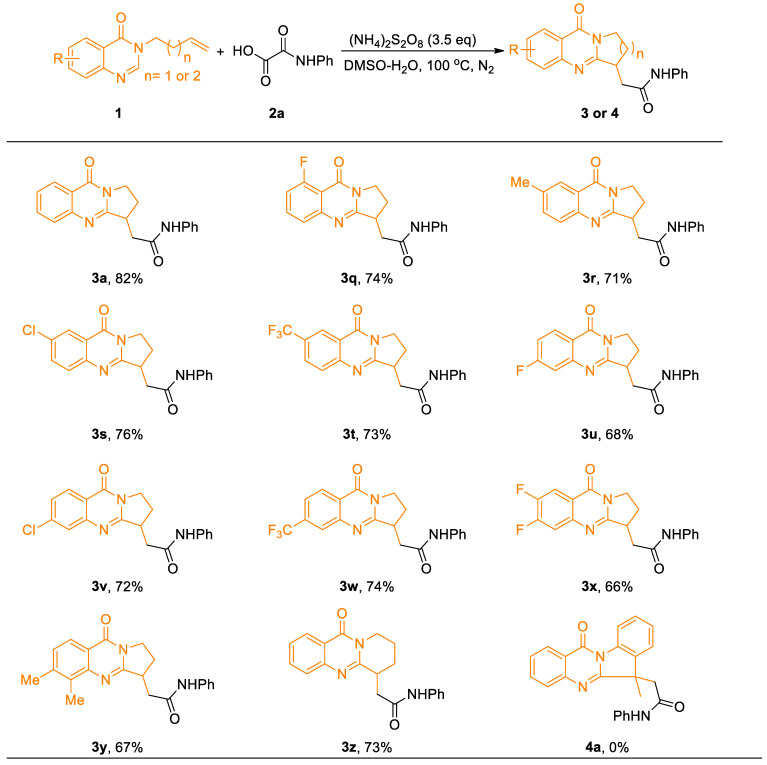
Preparation of **3a, 3q**–**3z**. Conditions: **1** (0.3 mmol, 1 eq), **2a** (0.6 mmol, 2 eq), (NH_4_)_2_S_2_O_8_ (1.05 mmol, 3.5 eq), DMSO-H_2_O (3 mL, *v*/*v* 100:1), 100 °C, under N_2_ atmosphere for 6–8 h. Isolated yields were given.

We then turned to investigate the reactions of **2a** with other quinazolinones, as shown in Scheme 3. We found these investigated quinazolinones substituted with several important functional groups, such as methyl (**3r**), fluoro (**3q** and **3u**), chloro (**3s** and **3v**), and trifluoromethyl (**3t** and **3w**) groups at the C5–C7 positions of the benzene ring were all compatible with the present reaction under standard conditions and provided the corresponding products in 66–82% yields. Furthermore, di-substituted quinazolinones also gave the expected products **3x** and **3y** in 66 and 67% yields, respectively. To our satisfaction, six-ring-fused quinazolinone product **3z** could also be obtained in a good yield. However, 3-(2-(prop-1-en-2-yl)phenyl)quinazolin-4(3*H*)-one failed to give the cyclization product (**4a**).

**Scheme 4:**
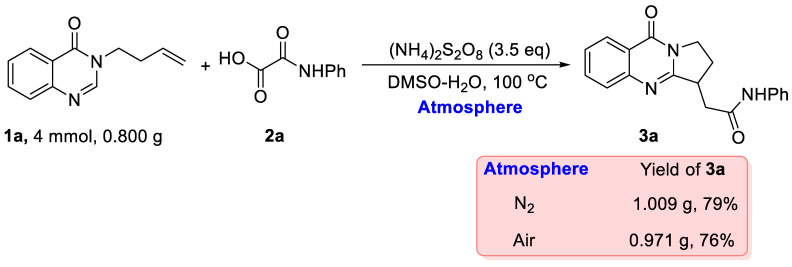
Gram-scale synthesis of **3a**.

The synthetic application of the present reaction was demonstrated via a gram-scale experiment, as shown in Scheme 4. The reaction of 3-(but-3-en-1-yl)quinazolin-4(3*H*)-one (**1a**, 4 mmol) with 2-oxo-2-(phenylamino)acetic acid **2a** could proceed well to provide the desired product **3a** in 79% isolated yield under standard reaction conditions. In addition, when the reaction was carried out in air atmosphere condition, a slightly decreased product was obtained in 76% yield.

**Scheme 5:**
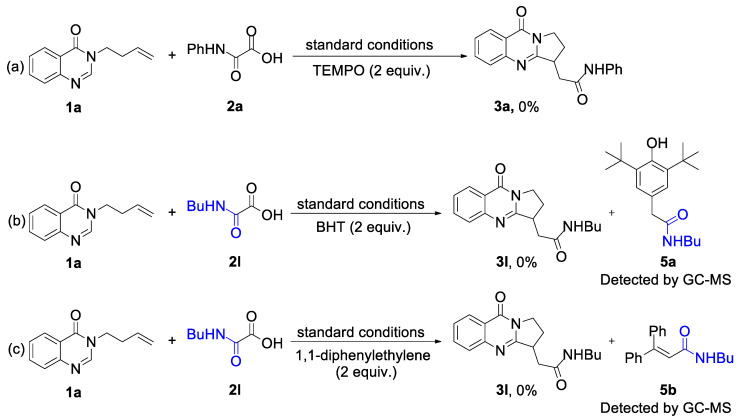
Control experiments. (**a**) The reaction of **1a** and **2a** in the presence of TEMPO. (**b**) The reaction of **1a** and **2a** in the presence of BHT. (**c**) The reaction of **1a** and **2a** in the presence of 1,1-diphenylethylene.

To better understand the reaction process of the present reaction, several control experiments were carried out, as shown in Scheme 5. First, the model reaction was performed under standard conditions with two equiv. of 2,2,6,6-tetramethylpiperidine-1-oxyl (TEMPO) as a radical scavenger, and no desired product **3a** was observed (Scheme 5a), indicating a free radical might be involved in the current transformation. Furthermore, a reaction of **1a** and **2l** was conducted with the addition of two equiv. of 2,6-di-tert-butyl-4-methylphenol (BHT) or 1,1-diphenylethylene, and **3l** was not observed. Meanwhile, radical-adducts **5a** and **5b** were detected through GC-MS analysis (Scheme 5b,c). These results implied that a carbamoyl radical might generate during the reaction process.

**Scheme 6:**
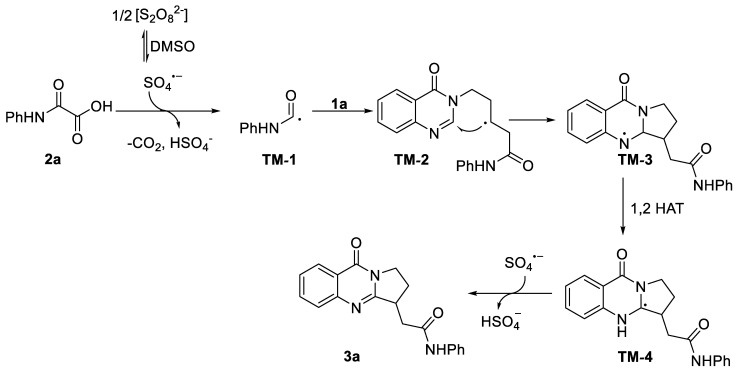
Possible mechanism.

Based on the control experiment and relevant reports [69,70,71,72], we speculated a possible reaction pathway, as shown in Scheme 6. Initially, compound **2a** might give a key carbamoyl radical **TM-1** through a successive oxidative, hydrogen atom transfer (HAT), and decarboxylative process in the presence of SO_4_^−•^, which can be generated via the heat-promoted homolytic cleavage of (NH_4_)_2_S_2_O_8_ in DMSO at high temperature. The radical **TM-1** further attacks the carbon–carbon double bond of **1a** to form a carbon radical intermediate **TM-2.** Then, the radical intermediate **TM-2** adds to the C=N bond to afford a nitrogen radical **TM-3**, which undergoes a 1,2-hydrogen atom transfer (1,2-HAT) process to deliver another carbon radical intermediate **TM-4**. Finally, the target product **3a** is generated through oxidative dehydrogenation of **TM-4** in the presence of radical anion SO_4_^−•^.

## 3. Experimental Section

### 3.1. General Information

Unless otherwise specified, all reagents and reaction solvents were obtained from commercial suppliers and used without further purification. The NMR spectra were recorded (Supplementary Materials Figures S1–S50) on an NMR spectrometer (Bruker, Rheinstetten, Germany, ^1^H NMR at 400 MHz, ^13^C NMR at 100 MHz and ^19^F NMR at 376 MHz, respectively). Chemical shifts were calibrated in ppm using residual CDCl_3_ as an internal reference (δ 7.26 ppm for ^1^H and 77.0 ppm for ^13^C). The high-resolution mass spectra (HRMS) were recorded on a spectrometer operating on ESI-TOF. Melting points were measured on a melting point apparatus and are uncorrected.

### 3.2. General Procedure for the Preparation of ***3***

To an oven-dried reaction vessel equipped with a magnetic stir bar was added quinazolin-4(3*H*)-one **1** (0.3 mmol, 1 eq.), oxamic acid **2** (0.6 mmol, 2 eq.), and (NH_4_)_2_S_2_O_8_ (1.05 mmol, 3.5 eq.) in DMSO-H_2_O mixed solvent (3 mL, *v*/*v* 100:1). The reaction mixture was stirred at 100 °C under N_2_ atmosphere for about 6–8 h, which was monitored by thin-layer chromatography (TLC). After completion, the reaction was allowed to cool to room temperature and then H_2_O (5 mL) was added to the mixture, which was further extracted with CH_2_Cl_2_ three times (10 mL × 3). The organic phase was then dried with anhydrous sodium sulfate and concentrated under vacuum. The residue was purified by flash column chromatography using a mixture of solvent consisting of petroleum ether and ethyl acetate as eluent (PE/EA 3:1–1:1) to obtain the desired product **3**.

### 3.3. Gram-Scale Synthesis of ***3a***

To an oven-dried reaction vessel equipped with a magnetic stir bar was added 3-(but-3-en-1-yl)quinazolin-4(3*H*)-one **1a** (4 mmol, 0.800 g), 2-oxo-2-(phenylamino)acetic acid **2a** (8 mmol, 1.320 g), and (NH_4_)_2_S_2_O_8_ (14 mmol, 3.195 g) in DMSO-H_2_O mixed solvent (40 mL, *v*/*v* 100:1). The reaction mixture was stirred at 100 °C under N_2_ atmosphere for about 6 h. After completion, the reaction was allowed to cool to room temperature and then H_2_O (30 mL) was added to the mixture, which was further extracted with CH_2_Cl_2_ three times (30 mL × 3). The organic phase was then dried with anhydrous sodium sulfate and concentrated under vacuum. The residue was purified by flash column chromatography using a mixture solvent consisting of petroleum ether and ethyl acetate as eluent (PE/EA 3:1–1:1) to obtain 1.009 g of **3a**.

### 3.4. Characterization Data of Products ***3a**–**3z***

2-(9-oxo-1,2,3,9-tetrahydropyrrolo

[2,1-b]quinazolin-3-yl)-*N*-phenylacetamide (**3a**): 

White solid; mp 172–173 °C; 78.5 mg (isolated yield 82%); ^1^H NMR (400 MHz, Chloroform-*d*) δ 9.86 (s, 1H), 8.30 (d, *J* = 7.9 Hz, 1H), 7.77 (t, *J* = 7.5 Hz, 1H), 7.70 (d, *J* = 8.0 Hz, 1H), 7.57 (d, *J* = 8.0 Hz, 2H), 7.49 (t, *J* = 7.5 Hz, 1H), 7.32 (t, *J* = 7.7 Hz, 2H), 7.09 (t, *J* = 7.3 Hz, 1H), 4.45–4.26 (m, 1H), 4.02–3.86 (m, 1H), 3.83–3.64 (m, 1H), 3.16 (dd, *J* = 15.1, 7.7 Hz, 1H), 2.73 (dd, *J* = 15.1, 4.3 Hz, 1H), 2.63 (dt, *J* = 13.8, 7.5 Hz, 1H), 2.08–1.97 (m, 1H); ^13^C NMR (100 MHz, Chloroform-*d*) δ 168.9, 161.3, 160.5, 148.2, 138.3, 134.5, 129.0, 126.8, 126.7, 126.4, 124.1, 120.7, 119.7, 44.8, 40.9, 40.0, 27.7. HRMS (ESI) *m*/*z* calcd for C_19_H_18_N_3_O_2_ [M + H]^+^: 320.1394; found: 320.1397.

2-(9-oxo-1,2,3,9-tetrahydropyrrolo

[2,1-b]quinazolin-3-yl)-*N*-(p-tolyl)acetamide (**3b**):

White solid; mp 167–168 °C; 82.9 mg (isolated yield 83%); ^1^H NMR (400 MHz, Chloroform-*d*) δ 9.64 (s, 1H), 8.29 (d, *J* = 7.6 Hz, 1H), 7.75 (t, *J* = 6.8 Hz, 1H), 7.68 (d, *J* = 7.7 H, 1H), 7.51–7.42 (m, 3H), 7.11 (d, *J* = 7.5 Hz, 2H), 4.34 (t, *J* = 10.4 Hz, 1H), 3.99–3.89 (m, 1H), 3.76–3.65 (m, 1H), 3.20–3.10 (m, 1H), 2.74–2.68 (m, 1H), 2.67–2.58 (m, 1H), 2.30 (s, 3H), 2.08–1.98 (m, 1H); ^13^C NMR (100 MHz, Chloroform-*d*) δ 168.8, 161.2, 160.6, 148.3, 135.7, 134.4, 133.7, 129.5, 126.7,126.6, 126.5, 120.7, 119.7, 44.8, 40.9, 39.9, 27.6, 20.8. HRMS (ESI) *m*/*z* calcd for C_20_H_20_N_3_O_2_ [M + H]^+^: 334.1550; found: 334.1552.

*N*-(4-fluorophenyl)-2-(9-oxo-1,2,3,9-tetrahydropyrrolo [2,1-b]quinazolin-3-yl)acetamide (**3c**):

White solid; mp 198–199 °C; 74.8 mg (isolated yield 74%); ^1^H NMR (400 MHz, Chloroform-*d*) δ 10.00 (s, 1H), 8.32 (d, *J* = 7.6 Hz, 1H), 7.79 (t, *J* = 7.5 Hz, 1H), 7.71 (d, *J* = 7.7 Hz, 1H), 7.58–7.48 (m, 3H), 7.02 (t, *J* = 8.3 Hz, 2H), 4.49–4.29 (m, 1H), 4.02–3.94 (m, 1H), 3.82–3.65 (m, 1H), 3.15 (dd, *J* = 14.9, 7.8 Hz, 1H), 2.77 (d, *J* = 14.8 Hz, 1H), 2.71–2.55 (m, 1H), 2.09–2.01 (m, 1H); ^13^C NMR (100 MHz, Chloroform-*d*) δ 168.9, 161.3, 160.5, 159.1 (d, *J*_C-F_ = 242.1 Hz), 148.1, 134.5, 134.4 (d, *J*_C-F_ = 2.4 Hz), 126.8, 126.7, 126.3, 121.3 (d, *J*_C-F_ = 7.8 Hz), 120.7, 115.6 (d, *J*_C-F_ = 22.3 Hz), 44.8, 40.8, 39.9, 27.6; ^19^F NMR (376 MHz, Chloroform-*d*) δ -118.24. HRMS (ESI) *m*/*z* calcd for C_19_H_17_FN_3_O_2_ [M + H]^+^: 338.1299; found: 338.1304.

*N*-(4-chlorophenyl)-2-(9-oxo-1,2,3,9-tetrahydropyrrolo [2,1-b]quinazolin-3-yl)acetamide (**3d**):

White solid; mp 215–216 °C; 76.3 mg (isolated yield 72%); ^1^H NMR (400 MHz, Chloroform-*d*) δ 10.16 (s, 1H), 8.32 (d, *J* = 7.9 Hz, 1H), 7.80 (t, *J* = 7.6 Hz, 1H), 7.71 (d, *J* = 8.0 Hz, 1H), 7.60–7.44 (m, 3H), 7.32–7.26 (m, 2H), 4.49–4.30 (m, 1H), 4.06–3.91 (m, 1H), 3.74 (d, *J* = 7.6 Hz, 1H), 3.15 (dd, *J* = 15.1, 8.0 Hz, 1H), 2.78 (d, *J* = 15.0 Hz, 1H), 2.72–2.57 (m, 1H), 2.11–2.00 (d, *J* = 21.4 Hz, 1H); ^13^C NMR (100 MHz, Chloroform-*d*) δ 169.0, 161.3, 160.5, 148.0, 137.0, 134.5, 129.0, 128.9, 126.9, 126.7, 126.3, 120.8, 120.7, 44.8, 40.8, 40.0, 27.7. HRMS (ESI) *m*/*z* calcd for C_19_H_17_ClN_3_O_2_ [M + H]^+^: 354.1004; found: 354.1006.

*N*-(4-bromophenyl)-2-(9-oxo-1,2,3,9-tetrahydropyrrolo [2,1-b]quinazolin-3-yl)acetamide (**3e**):

White solid; mp 213–214 °C; 85.8 mg (isolated yield 72%); ^1^H NMR (400 MHz, Chloroform-*d*) δ 10.12 (s, 1H), 8.30 (d, *J* = 7.9 Hz, 1H), 7.77 (t, *J* = 7.6 Hz, 1H), 7.67 (d, *J* = 8.0 Hz, 1H), 7.52–7.45 (m, 3H), 7.42 (d, *J* = 8.7 Hz, 2H), 4.36 (t, *J* = 10.4 Hz, 1H), 4.02–3.89 (m, 1H), 3.80–3.66 (m, 1H), 3.14 (dd, *J* = 15.1, 8.0 Hz, 1H), 2.72 (dd, *J* = 15.1, 4.1 Hz, 1H), 2.66–2.57 (m, *1H*), 2.07–1.98 (m, 1H); ^13^C NMR (100 MHz, Chloroform-*d*) δ 169.0, 161.3, 160.4, 148.0, 137.5, 134.5, 131.9, 126.8, 126.7, 126.3, 121.2, 120.7, 116.5, 44.8, 40.8, 40.1, 27.7. HRMS (ESI) *m*/*z* calcd for C_19_H_17_BrN_3_O_2_ [M + H]^+^: 398.0499; found: 398.0494.

2-(9-oxo-1,2,3,9-tetrahydropyrrolo [2,1-b]quinazolin-3-yl)-N-(4-phenoxyphenyl)acetamide (**3f**):

White solid; mp 197–198 °C; 80.2 mg (isolated yield 65%); ^1^H NMR (400 MHz, Chloroform-*d*) δ 9.89 (s, 1H), 8.31 (d, *J* = 7.9 Hz, 1H), 7.77 (t, *J* = 7.6 Hz, 1H), 7.69 (d, *J* = 8.0 Hz, 1H), 7.55 (d, *J* = 8.3 Hz, 2H), 7.49 (t, *J* = 7.5 Hz, 1H), 7.31 (t, *J* = 7.6 Hz, 2H), 7.08 (t, *J* = 7.3 Hz, 1H), 7.04–6.86 (m, 4H), 4.37 (t, *J* = 10.5 Hz, 1H), 3.97 (q, *J* = 11.1, 10.7 Hz, 1H), 3.81–3.67 (m, 1H), 3.16 (dd, *J* = 15.1, 7.7 Hz, 1H), 2.75 (dd, *J* = 15.1, 3.6 Hz, 1H), 2.70–2.58 (m, 1H), 2.10–2.00 (m, 1H); ^13^C NMR (100 MHz, Chloroform-*d*) δ 168.8, 161.3, 160.5, 157.5, 153.2, 148.1, 134.5, 133.9, 129.7, 126.8, 126.7, 126.4, 123.0, 121.3, 120.7, 119.7, 118.3, 44.8, 40.9, 39.9, 27.6. HRMS (ESI) *m*/*z* calcd for C_25_H_22_N_3_O_3_ [M + H]^+^: 412.1656; found: 412.1662.

2-(9-oxo-1,2,3,9-tetrahydropyrrolo [2,1-b]quinazolin-3-yl)-*N*-(4-(trifluoromethyl)phenyl)acetamide (**3g**):

White solid; mp 224–225 °C; 88.3 mg (isolated yield 76%); ^1^H NMR (400 MHz, Chloroform-*d*) δ 10.48 (s, 1H), 8.29 (d, *J* = 7.9 Hz, 1H), 7.77 (t, *J* = 7.5 Hz, 1H), 7.74–7.64 (m, 3H), 7.56 (d, *J* = 8.1 Hz, 2H), 7.49 (t, *J* = 7.5 Hz, 1H), 4.44–4.29 (m, 1H), 4.01–3.86 (m, 1H), 3.79–3.67 (m, 1H), 3.17 (dd, *J* = 15.1, 8.1 Hz, 1H), 2.76 (dd, *J* = 15.1, 3.4 Hz, 1H), 2.63 (dt, *J* = 14.4, 7.6 Hz, 1H), 2.09–1.98 (m, 1H); ^13^C NMR (100 MHz, Chloroform-*d*) δ 169.3, 161.4, 160.4, 147.8, 141.5, 134.6, 127.0, 126.7, 126.3, 126.1, 124.1 (q, *J*_C-F_ = 269.8 Hz), 125.7 (q, *J*_C-F_ = 34.0 Hz), 120.6, 119.2, 44.8, 40.7, 40.1, 27.7; ^19^F NMR (376 MHz, Chloroform-*d*) δ -62.07. HRMS (ESI) *m*/*z* calcd for C_20_H_17_F_3_N_3_O_2_ [M + H]^+^: 388.1267; found: 388.1275.

2-(9-oxo-1,2,3,9-tetrahydropyrrolo [2,1-b]quinazolin-3-yl)-*N*-(4-((trifluoromethyl)thio)phenyl)acetamide (**3h**):

White solid; mp 206–207 °C; 78.0 mg (isolated yield 62%); ^1^H NMR (400 MHz, Chloroform-*d*) δ 10.51 (s, 1H), 8.32 (d, *J* = 7.8 Hz, 1H), 7.81 (t, *J* = 7.6 Hz, 1H), 7.74–7.65 (m, 3H), 7.61 (d, *J* = 7.9 Hz, 2H), 7.52 (t, *J* = 7.6 Hz, 1H), 4.44–4.33 (m, 1H), 4.02–3.93 (m, 1H), 3.78–3.66 (m, 1H), 3.17 (dd, *J* = 15.0, 8.1 Hz, 1H), 2.78 (d, *J* = 15.2 Hz, 1H), 2.71–2.58 (m, 1H), 2.11–1.98 (m, 1H); ^13^C NMR (100 MHz, Chloroform-*d*) δ 169.3, 161.4, 160.4, 147.8, 141.0, 137.5, 134.6, 129.5 (d, *J*_C-F_ = 306.5 Hz), 127.0, 126.8, 126.1, 120.6, 120.1, 118.4, 44.8, 40.8, 40.2, 27.7; ^19^F NMR (376 MHz, Chloroform-*d*) δ −43.35. HRMS (ESI) *m*/*z* calcd for C_20_H_17_F_3_N_3_O_2_S [M + H]^+^: 420.0988; found: 420.0992.

*N*-(2-bromophenyl)-2-(9-oxo-1,2,3,9-tetrahydropyrrolo [2,1-b]quinazolin-3-yl)acetamide (**3i**):

White solid; mp 197–198 °C; 69.1 mg (isolated yield 58%); ^1^H NMR (400 MHz, Chloroform-*d*) δ 8.55 (s, 1H), 8.36–8.15 (m, 2H), 7.78–7.65 (m, 2H), 7.55–7.41 (m, 2H), 7.35–7.27 (m, 1H), 7.03–6.92 (m, 1H), 4.48–4.24 (m, 1H), 4.11–3.90 (m, 1H), 3.88–3.68 (m, 1H), 3.35–3.14 (m, 1H), 3.00–2.80 (m, 1H), 2.76–2.59 (m, 1H), 2.16–1.99 (m, 1H); ^13^C NMR (100 MHz, Chloroform-*d*) δ 168.9, 160.6, 148.4, 135.6, 134.3, 132.4, 128.2, 126.7, 126.6, 126.5, 125.7, 123.0, 120.7, 114.1, 44.9, 40.7, 39.3, 27.0. HRMS (ESI) *m*/*z* calcd for C_19_H_17_BrN_3_O_2_ [M + H]^+^: 398.0499; found: 398.0495.

2-(9-oxo-1,2,3,9-tetrahydropyrrolo

[2,1-b]quinazolin-3-yl)-*N*-(m-tolyl)acetamide (**3j**):

White solid; mp 238–239 °C; 63.9 mg (isolated yield 64%); ^1^H NMR (400 MHz, Chloroform-*d*) δ 9.71 (s, 1H), 8.39 (d, *J* = 7.9 Hz, 1H), 7.85 (t, *J* = 7.5 Hz, 1H), 7.79 (d, *J* = 8.0 Hz, 1H), 7.57 (t, *J* = 7.3 Hz, 1H), 7.52 (s, 1H), 7.39 (d, *J* = 7.9 Hz, 1H), 7.28 (t, *J* = 7.7 Hz, 1H), 6.99 (d, *J* = 7.2 Hz, 1H), 4.44 (t, *J* = 10.5 Hz, 1H), 4.04 (q, *J* = 10.7 Hz, 1H), 3.78 (d, *J* = 18.6 Hz, 1H), 3.23 (dd, *J* = 14.9, 7.2 Hz, 1H), 2.83 (d, *J* = 14.7 Hz, 1H), 2.77–2.66 (m, 1H), 2.41 (s, 3H), 2.17–2.06 (m, 1H); ^13^C NMR (100 MHz, Chloroform-*d*) δ 168.8, 161.3, 160.5, 148.2, 138.9, 138.2, 134.4, 128.8, 126.8, 126.7, 126.4, 124.9, 120.7, 120.4, 116.7, 44.8, 40.9, 40.0, 27.6, 21.5. HRMS (ESI) *m*/*z* calcd for C_20_H_20_N_3_O_2_ [M + H]^+^: 334.1550; found: 334.1557.

*N*-(3-bromophenyl)-2-(9-oxo-1,2,3,9-tetrahydropyrrolo [2,1-b]quinazolin-3-yl)acetamide (**3k**):

White solid; mp 234–235 °C; 79.8 mg (isolated yield 67%); ^1^H NMR (400 MHz, Chloroform-*d*) δ 10.21 (s, 1H), 8.31 (d, *J* = 7.8 Hz, 1H), 7.88 (s, 1H), 7.79 (t, *J* = 7.6 Hz, 1H), 7.70 (d, *J* = 8.0 Hz, 1H), 7.49 (dd, *J* = 20.1, 7.6 Hz, 2H), 7.25–7.14 (m, 2H), 4.47–4.25 (m, 1H), 4.09–3.89 (m, 1H), 3.78–3.61 (m, 1H), 3.15 (dd, *J* = 15.0, 8.2 Hz, 1H), 2.73 (dd, *J* = 15.1, 3.4 Hz, 1H), 2.64 (dt, *J* = 14.1, 7.7 Hz, 1H), 2.08–1.98 (m, 1H); ^13^C NMR (100 MHz, Chloroform-*d*) δ 169.1, 161.3, 160.5, 148.0, 139.7, 134.6, 130.3, 127.0, 126.9, 126.7, 126.3, 122.6, 122.6, 120.7, 118.0, 44.8, 40.8, 40.1, 27.8. HRMS (ESI) *m*/*z* calcd for C_19_H_17_BrN_3_O_2_ [M + H]^+^: 398.0499; found: 398.0494.

*N*-butyl-2-(9-oxo-1,2,3,9-tetrahydropyrrolo

[2,1-b]quinazolin-3-yl)acetamide (**3l**): 

White solid; mp 167–168 °C; 69.1 mg (isolated yield 77%); ^1^H NMR (400 MHz, Chloroform-*d*) δ 8.29 (d, *J* = 7.9 Hz, 1H), 7.74 (t, *J* = 7.6 Hz, 1H), 7.63 (d, *J* = 8.0 Hz, 1H), 7.46 (t, *J* = 7.5 Hz, 1H), 6.85 (s, 1H), 4.33 (t, *J* = 10.5 Hz, 1H), 4.01–3.91 (m, 1H), 3.74–3.57 (m, 1H), 3.31- 3.24 (m, 2H), 2.97 (dd, *J* = 14.9, 6.2 Hz, 1H), 2.59 (dd, *J* = 14.6, 6.2 Hz, 2H), 2.02 (d, *J* = 11.6 Hz, 1H), 1.52–1.44 (m 2H), 1.34 (dt, *J* = 14.8, 7.4 Hz, 2H), 0.89 (t, *J* = 7.2 Hz, 3H); ^13^C NMR (100 MHz, Chloroform-*d*) δ 170.4, 161.1, 160.7, 148.5, 134.2, 126.6, 126.5, 120.7, 44.8, 41.0, 39.3, 38.5, 31.6, 27.1, 20.1, 13.7. HRMS (ESI) *m*/*z* calcd for C_17_H_22_N_3_O_2_ [M + H]^+^: 300.1707; found: 300.1714.

*N*-cyclopentyl-2-(9-oxo-1,2,3,9-tetrahydropyrrolo

[2,1-b]quinazolin-3-yl)acetamide (**3m**):

White solid; mp 206–207 °C; 67.2 mg (isolated yield 72%); ^1^H NMR (400 MHz, Chloroform-*d*) δ 8.29 (d, *J* = 7.8 Hz, 1H), 7.73 (t, *J* = 7.4 Hz, 1H), 7.62 (d, *J* = 8.1 Hz, 1H), 7.46 (t, *J* = 7.4 Hz, 1H), 7.02 (s, 1H), 4.32 (t, *J* = 10.2 Hz, 1H), 4.24–4.15 (m, 1H), 4.00–3.90 (m, 1H), 3.67–3.59 (m, 1H), 2.92 (dd, *J* = 14.9, 6.4 Hz, 1H), 2.68–2.48 (m, 2H), 2.10–1.86 (m, 4H), 1.71- 1.53 (m, 4H), 1.45–1.34 (m, 1H); ^13^C NMR (100 MHz, Chloroform-*d*) δ 170.0, 161.1, 160.7, 148.5, 134.3, 126.6, 126.5, 120.7, 51.3, 44.8, 41.1, 38.7, 33.1, 27.2, 23.7. HRMS (ESI) *m*/*z* calcd for C_18_H_22_N_3_O_2_ [M + H]^+^: 312.1707; found: 312.1712.

*N*-(adamantan-1-yl)-2-(9-oxo-1,2,3,9-tetrahydropyrrolo [2,1-b]quinazolin-3-yl)acetamide (**3n**):

White solid; mp 85–86 °C; 88.2 mg (isolated yield 78%); ^1^H NMR (400 MHz, Chloroform-*d*) δ 8.29 (d, *J* = 7.9 Hz, 1H), 7.73 (t, *J* = 7.6 Hz, 1H), 7.64 (d, *J* = 8.1 Hz, 1H), 7.46 (t, *J* = 7.5 Hz, 1H), 6.46 (s, 1H), 4.32 (t, *J* = 10.4 Hz, 1H), 4.02–3.89 (m, 1H), 3.65–3.58 (m, 1H), 2.90 (dd, *J* = 14.8, 5.9 Hz, 1H), 2.65–2.54 (m, 1H), 2.49 (dd, *J* = 14.8, 6.3 Hz, 1H), 2.11–1.93 (m, 10H), 1.70–1.59 (m, 6H); ^13^C NMR (100 MHz, Chloroform-*d*) δ 169.6, 161.1, 160.7, 148.7, 134.2, 126.6, 126.5, 126.4, 120.7, 52.0, 44.8, 41.6, 41.2, 39.6, 36.3, 29.4, 27.1. HRMS (ESI) *m*/*z* calcd for C_23_H_28_N_3_O_2_ [M + H]^+^: 378.2176; found: 378.2179.

*N*-benzyl-2-(9-oxo-1,2,3,9-tetrahydropyrrolo

[2,1-b]quinazolin-3-yl)acetamide (**3o**):

White solid; mp 174–175 °C; 65.0 mg (isolated yield 65%); ^1^H NMR (400 MHz, Chloroform-*d*) δ 8.26 (d, *J* = 7.9 Hz, 1H), 7.67 (t, *J* = 7.5 Hz, 1H), 7.49–7.34 (m, 3H), 7.26 (s, 5H), 4.56–4.37 (m, 2H), 4.37–4.26 (m, 1H), 4.01–3.86 (m, 1H), 3.74–3.61 (m, 1H), 3.00 (dd, *J* = 15.0, 6.5 Hz, 1H), 2.72–2.54 (m, 2H), 2.11–1.98 (m, 1H); ^13^C NMR (100 MHz, Chloroform-*d*) δ 170.4, 160.8, 160.7, 148.5, 138.1, 134.1, 128.7, 127.8, 127.5, 126.6, 126.5, 126.5, 120.7, 44.7, 40.9, 38.5, 27.2. HRMS (ESI) *m*/*z* calcd for C_20_H_20_N_3_O_2_ [M + H]^+^: 334.1550; found: 334.1557.

2-(8-fluoro-9-oxo-1,2,3,9-tetrahydropyrrolo [2,1-b]quinazolin-3-yl)-*N*-phenylacetamide (**3q**):

White solid; mp 202–203 °C; 74.8 mg (isolated yield 74%); ^1^H NMR (400 MHz, Chloroform-*d*) δ 9.45 (s, 1H), 7.68 (q, *J* = 8.1 Hz, 1H), 7.55 (d, *J* = 7.9 Hz, 2H), 7.47 (d, *J* = 8.2 Hz, 1H), 7.32 (t, *J* = 7.6 Hz, 2H), 7.17–7.05 (m, 2H), 4.42–4.27 (m, 1H), 4.01–3.86 (m, 1H), 3.71 (d, *J* = 15.0 Hz, 1H), 3.15 (dd, *J* = 15.1, 7.2 Hz, 1H), 2.73 (dd, *J* = 15.1, 4.7 Hz, 1H), 2.67–2.56 (m, 1H), 2.09–1.98 (m, 1H); ^13^C NMR (100 MHz, Chloroform-*d*) δ 168.7, 162.8, 162.1, 158.9 (d, *J*_C-F_ = 249.5 Hz), 150.6, 138.1, 134.8 (d, *J*_C-F_ = 10.4 Hz), 129.0, 124.2, 122.4 (d, *J*_C-F_ = 4.2 Hz), 119.7, 113.3 (d, *J*_C-F_ = 20.8 Hz), 110.5 (d, *J*_C-F_ = 6.9 Hz), 44.8, 40.9, 39.7, 27.3; ^19^F NMR (376 MHz, Chloroform-*d*) δ -110.23. HRMS (ESI) *m*/*z* calcd for C_19_H_17_FN_3_O_2_ [M + H]^+^: 338.1299; found: 338.1292.

2-(7-methyl-9-oxo-1,2,3,9-tetrahydropyrrolo [2,1-b]quinazolin-3-yl)-*N*-phenylacetamide (**3r**):

White solid; mp 217–218 °C; 71.0 mg (isolated yield 71%); ^1^H NMR (400 MHz, Chloroform-*d*) δ 10.03 (s, 1H), 8.10 (s, 1H), 7.65–7.53 (m, 4H), 7.34 (d, *J* = 6.6 Hz, 2H), 7.10 (t, *J* = 6.7 Hz, 1H), 4.37 (t, *J* = 10.7 Hz, 1H), 3.95 (q, *J* = 11.3, 10.6 Hz, 1H), 3.71 (d, *J* = 17.3 Hz, 1H), 3.25–3.10 (m, 1H), 2.73 (d, *J* = 15.2 Hz, 1H), 2.63 (dt, *J* = 15.0, 8.0 Hz, 1H), 2.50 (s, 3H), 2.07–1.97 (m, 1H); ^13^C NMR (100 MHz, Chloroform-*d*) δ 169.0, 160.6, 160.4, 146.2, 138.4, 137.0, 135.9, 129.0, 126.2, 126.1, 124.1, 120.5, 119.6, 44.7, 40.8, 40.2, 27.8, 21.3. HRMS (ESI) *m*/*z* calcd for C_20_H_20_N_3_O_2_ [M + H]^+^: 334.1550; found: 334.1556.

2-(7-chloro-9-oxo-1,2,3,9-tetrahydropyrrolo [2,1-b]quinazolin-3-yl)-*N*-phenylacetamide (**3s**):

White solid; mp 238–239 °C; 80.5 mg (isolated yield 76%); ^1^H NMR (400 MHz, Chloroform-*d*) δ 9.36 (s, 1H), 8.27 (s, 1H), 7.71 (d, *J* = 8.7 Hz, 1H), 7.64 (d, *J* = 8.6 Hz, 1H), 7.54 (d, *J* = 7.9 Hz, 2H), 7.33 (t, *J* = 7.5 Hz, 2H), 7.11 (t, *J* = 7.4 Hz, 1H), 4.49–4.31 (m, 1H), 4.05–3.93 (m, 1H), 3.81–3.67 (m, 1H), 3.15 (dd, *J* = 15.2, 7.2 Hz, 1H), 2.75 (dd, *J* = 15.2, 4.6 Hz, 1H), 2.71–2.59 (m, 1H), 2.12–2.02 (m, 1H); ^13^C NMR (100 MHz, Chloroform-*d*) δ 168.7, 161.5, 159.5, 146.8, 138.1, 134.8, 132.6, 129.1, 128.1, 126.1, 124.3, 121.8, 119.7, 44.9, 40.9, 39.7, 27.5. HRMS (ESI) *m*/*z* calcd for C_19_H_17_ClN_3_O_2_ [M + H]^+^: 354.1004; found: 354.1002.

2-(9-oxo-7-(trifluoromethyl)-1,2,3,9-tetrahydropyrrolo [2,1-b]quinazolin-3-yl)-*N*-phenylacetamide (**3t**):

White solid; mp 191–192 °C; 84.8 mg (isolated yield 73%); ^1^H NMR (400 MHz, Chloroform-*d*) δ 9.10 (s, 1H), 8.57 (s, 1H), 7.94 (d, *J* = 8.5 Hz, 1H), 7.76 (d, *J* = 8.5 Hz, 1H), 7.52 (d, *J* = 7.8 Hz, 2H), 7.31 (t, *J* = 7.6 Hz, 2H), 7.10 (t, *J* = 7.3 Hz, 1H), 4.46–4.28 (m, 1H), 4.04–3.93 (m, 1H), 3.74 (d, *J* = 15.5 Hz, 1H), 3.17 (dd, *J* = 15.3, 6.6 Hz, 1H), 2.76 (dd, *J* = 15.3, 5.3 Hz, 1H), 2.73–2.58 (m, 1H), 2.12–2.01 (m, 1H); ^13^C NMR (100 MHz, Chloroform-*d*) δ 168.6, 163.3, 159.8, 150.6, 137.9, 130.6 (q, *J*_C-F_ = 3.2 Hz), 129.0, 128.6 (q, *J*_C-F_ = 33.3 Hz), 127.5, 124.5 (q, *J*_C-F_ = 4.0 Hz), 124.3, 123.6 (q, *J*_C-F_ = 270.5 Hz), 120.7, 119.7, 45.0, 41.0, 39.4, 27.2; ^19^F NMR (376 MHz, Chloroform-*d*) δ -62.27. HRMS (ESI) *m*/*z* calcd for C_20_H_17_F_3_N_3_O_2_ [M + H]^+^: 388.1267; found: 388.1261.

2-(6-fluoro-9-oxo-1,2,3,9-tetrahydropyrrolo [2,1-b]quinazolin-3-yl)-*N*-phenylacetamide (**3u**):

White solid; mp 181–182 °C; 68.8 mg (isolated yield 68%); ^1^H NMR (400 MHz, Chloroform-*d*) δ 9.36 (s, 1H), 8.42–8.18 (m, 1H), 7.53 (d, *J* = 7.9 Hz, 2H), 7.32 (t, *J* = 7.7 Hz, 3H), 7.18 (t, *J* = 8.4 Hz, 1H), 7.10 (t, *J* = 7.3 Hz, 1H), 4.43–4.27 (m, 1H), 4.00–3.89 (m, 1H), 3.84–3.66 (m, 1H), 3.15 (dd, *J* = 15.2, 6.9 Hz, 1H), 2.73 (dd, *J* = 15.2, 5.1 Hz, 1H), 2.67–2.58 (m, 1H), 2.13–1.99 (m, 1H); ^13^C NMR (100 MHz, Chloroform-*d*) δ 168.7, 166.4 (d, *J*_C-F_ = 252.9 Hz), 162.5, 159.9, 150.6 (d, *J*_C-F_ = 12.8 Hz), 138.0, 129.3 (d, *J*_C-F_ = 10.6 Hz), 129.0, 124.2, 119.7, 117.4, 115.4 (d, *J*_C-F_ = 23.3Hz), 112.0 (d, *J*_C-F_ = 22.0Hz), 44.8, 40.9, 39.6, 27.4; ^19^F NMR (376 MHz, Chloroform-*d*) δ -103.09. HRMS (ESI) *m*/*z* calcd for C_19_H_17_FN_3_O_2_ [M + H]^+^: 338.1299; found: 338.1303.

2-(6-chloro-9-oxo-1,2,3,9-tetrahydropyrrolo [2,1-b]quinazolin-3-yl)-*N*-phenylacetamide (**3v**):

White solid; mp 187–188 °C; 76.3 mg (isolated yield 72%); ^1^H NMR (400 MHz, Chloroform-*d*) δ 9.24 (s, 1H), 8.21 (d, *J* = 8.5 Hz, 1H), 7.66 (s, 1H), 7.54 (d, *J* = 7.9 Hz, 2H), 7.42 (d, *J* = 8.5 Hz, 1H), 7.33 (t, *J* = 7.7 Hz, 2H), 7.11 (t, *J* = 7.4 Hz, 1H), 4.41–4.27 (m, 1H), 4.02–3.90 (m, 1H), 3.81–3.68 (m, 1H), 3.15 (dd, *J* = 15.2, 6.9 Hz, 1H), 2.73 (dd, *J* = 15.2, 5.1 Hz, 1H), 2.69–2.59 (m, 1H), 2.09–2.00 (m, 1H); ^13^C NMR (100 MHz, Chloroform-*d*) δ 168.7, 162.5, 160.0, 149.4, 140.6, 138.0, 129.1, 128.0, 127.3, 126.1, 124.3, 119.7, 119.2, 44.9, 40.9, 39.6, 27.4. HRMS (ESI) *m*/*z* calcd for C_19_H_17_ClN_3_O_2_ [M + H]^+^: 354.1004; found: 354.1007.

2-(9-oxo-6-(trifluoromethyl)-1,2,3,9-tetrahydropyrrolo [2,1-b]quinazolin-3-yl)-*N*-phenylacetamide (**3w**):

White solid; mp 203–204 °C; 85.9 mg (isolated yield 74%); ^1^H NMR (400 MHz, Chloroform-*d*) δ 8.91 (s, 1H), 8.39 (d, *J* = 8.2 Hz, 1H), 7.92 (s, 1H), 7.66 (d, *J* = 8.2 Hz, 1H), 7.52 (d, *J* = 7.8 Hz, 2H), 7.31 (t, *J* = 7.4 Hz, 2H), 7.10 (t, *J* = 7.2 Hz, 1H), 4.45–4.30 (m, 1H), 4.02–3.94 (m, 1H), 3.77 (d, *J* = 8.4 Hz, 1H), 3.18 (dd, *J* = 15.4, 6.1 Hz, 1H), 2.76 (dd, *J* = 15.3, 5.7 Hz, 1H), 2.72–2.60 (m, 1H), 2.17–2.04 (m, 1H); ^13^C NMR (100 MHz, Chloroform-*d*) δ 168.6, 162.5, 159.8, 148.5, 137.9, 135.8 (q, *J*_C-F_ = 32.8 Hz), 129.0, 127.7, 124.4, 124.1 (q, *J*_C-F_ = 3.9 Hz), 123.3 (q, *J*_C-F_ = 271.3 Hz), 123.0, 122.6 (q, *J*_C-F_ = 3.4 Hz), 119.7, 45.0, 40.8, 39.3, 27.1; ^19^F NMR (376 MHz, Chloroform-*d*) δ −63.14. HRMS (ESI) *m*/*z* calcd for C_20_H_17_F_3_N_3_O_2_ [M + H]^+^: 388.1267; found: 388.1270.

2-(6,7-difluoro-9-oxo-1,2,3,9-tetrahydropyrrolo [2,1-b]quinazolin-3-yl)-*N*-phenylacetamide (**3x**):

White solid; mp 220–221 °C; 70.3 mg (isolated yield 66%); ^1^H NMR (400 MHz, Chloroform-*d*) δ 8.94 (s, 1H), 8.05 (t, *J* = 9.2 Hz, 1H), 7.51 (d, *J* = 7.9 Hz, 2H), 7.45 (dd, *J* = 10.4, 7.1 Hz, 1H), 7.33 (t, *J* = 7.7 Hz, 2H), 7.11 (t, *J* = 7.3 Hz, 1H), 4.43–4.27 (m, 1H), 4.04–3.90 (m, 1H), 3.81–3.68 (m, 1H), 3.14 (dd, *J* = 15.3, 6.5 Hz, 1H), 2.75 (dd, *J* = 15.3, 5.4 Hz, 1H), 2.69–2.61 (m, 1H), 2.13–2.02 (m, 1H); ^13^C NMR (100 MHz, Chloroform-*d*) δ 168.6, 162.0, 159.2 (d, *J*_C-F_ = 2.5 Hz), 154.8 (dd, *J*_C-F_ = 256.7 Hz, 14.8 Hz), 147.2 (dd, *J*_C-F_ = 205.7 Hz, 13.8 Hz), 137.9, 129.1, 124.4, 119.7, 117.7 (d, *J*_C-F_ = 7.6 Hz), 114.5, 114.3, 114.0 (dd, *J*_C-F_ = 18.8 Hz, 1.7 Hz), 45.0, 40.9, 39.4, 27.3; ^19^F NMR (376 MHz, Chloroform-*d*) δ -126.08 (d, *J* = 21.6 Hz), -136.11 (d, *J* = 21.6 Hz). HRMS (ESI) *m*/*z* calcd for C_19_H_16_F_2_N_3_O_2_ [M + H]^+^: 356.1205; found: 356.1209.

2-(5,6-dimethyl-9-oxo-1,2,3,9-tetrahydropyrrolo [2,1-b]quinazolin-3-yl)-*N*-phenylacetamide (**3y**):

White solid; mp 233–234 °C; 69.8 mg (isolated yield 67%); ^1^H NMR (400 MHz, Chloroform-*d*) δ 9.21 (s, 1H), 8.05 (d, *J* = 8.1 Hz, 1H), 7.50 (d, *J* = 7.9 Hz, 2H), 7.35–7.24 (m, 3H), 7.10 (t, *J* = 7.3 Hz, 1H), 4.44–4.25 (m, 1H), 4.01–3.85 (m, 1H), 3.79–3.70 (m, 1H), 3.17 (dd, *J* = 15.1, 7.2 Hz, 1H), 2.75 (dd, *J* = 15.1, 4.7 Hz, 1H), 2.70–2.57 (m, 1H), 2.51 (s, 3H), 2.42 (s, 3H), 2.09–1.93 (m, 1H); ^13^C NMR (100 MHz, Chloroform-*d*) δ 169.3, 161.1, 159.6, 146.8, 143.4, 137.9, 132.8, 128.9, 128.6, 124.3, 123.4, 120.3, 118.6, 44.5, 41.0, 39.8, 27.6, 21.0, 13.5. HRMS (ESI) *m*/*z* calcd for C_21_H_22_N_3_O_2_ [M + H]^+^: 348.1707; found: 348.1708.

2-(11-oxo-7,8,9,11-tetrahydro-6H-pyrido

[2,1-b]quinazolin-6-yl)-*N*-phenylacetamide (**3z**):

White solid; mp 122–123 °C; 73.0 mg (isolated yield 73%); ^1^H NMR (400 MHz, Chloroform-*d*) δ 9.40 (s, 1H), 8.27 (d, *J* = 8.1 Hz, 1H), 7.75 (t, *J* = 7.6 Hz, 1H), 7.64 (d, *J* = 8.1 Hz, 1H), 7.48 (dd, *J* = 19.9, 7.9 Hz, 4H), 7.29 (d, *J* = 7.7 Hz, 1H), 7.06 (t, *J* = 7.3 Hz, 1H), 4.57–4.37 (m, 1H), 3.81 (t, *J* = 13.8 Hz, 1H), 3.34 (d, *J* = 11.1 Hz, 1H), 3.18 (dd, *J* = 14.2, 7.5 Hz, 1H), 2.77 (dd, *J* = 14.2, 3.5 Hz, 1H), 2.34–2.21 (m, 1H), 2.08–1.97 (m, 2H), 1.76–1.64 (m, 1H); ^13^C NMR (100 MHz, Chloroform-*d*) δ 170.0, 161.5, 157.7, 146.4, 138.2, 134.4, 129.0, 127.0, 126.6, 126.1, 123.9, 120.3, 119.5, 40.8, 40.6, 37.9, 26.0, 20.7. HRMS (ESI) m/z calcd for C_20_H_20_N_3_O_2_ [M + H]^+^: 334.1550; found: 334.1553.

## 4. Conclusions

In summary, we have developed a convenient method for the incorporation of an amide group to 2,3-fused quinazolin-4(3*H*)-ones via the radical cascade reaction of N3-alkenyl-tethered quinazolinones with readily available oxamic acids as amide sources. The reaction was performed well under transition-metal-free conditions with wide functional group compatibility, making it an attractive method for the construction of amide-containing quinazolinone derivatives.

## Figures and Tables

**Table 1:** Optimization of reaction conditions ^a^.

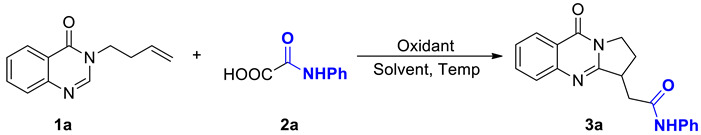				
Entry	Oxidant	Solvent	Temp	Yield of 3a ^b^
1	(NH_4_)_2_S_2_O_8_	DMSO	80	42%
2	K_2_S_2_O_8_	DMSO	80	18%
3	Na_2_S_2_O_8_	DMSO	80	24%
4	(NH_4_)_2_S_2_O_8_	H_2_O	80	27%
5	(NH_4_)_2_S_2_O_8_	DMSO/H_2_O (500:1)	80	43%
6	(NH_4_)_2_S_2_O_8_	DMSO/H_2_O (100:1)	80	57%
7	(NH_4_)_2_S_2_O_8_	DMSO/H_2_O (10:1)	80	46%
8	(NH_4_)_2_S_2_O_8_	DMSO/H_2_O (100:1)	90	64%
9	(NH_4_)_2_S_2_O_8_	DMSO/H_2_O (100:1)	100	76%
10	(NH_4_)_2_S_2_O_8_	DMSO/H_2_O (100:1)	110	73%
11	(NH_4_)_2_S_2_O_8_	DMSO/H_2_O (100:1)	70	16%
12 ^c^	(NH_4_)_2_S_2_O_8_	DMSO/H_2_O (100:1)	100	67%
13 ^d^	(NH_4_)_2_S_2_O_8_	DMSO/H_2_O (100:1)	100	82%
14 ^e^	(NH_4_)_2_S_2_O_8_	DMSO/H_2_O (100:1)	100	80%
15	None	DMSO/H_2_O (100:1)	100	0%

^a^ General conditions, unless otherwise noted: **1a** (0.3 mmol, 1 equiv.), **2a** (0.6 mmol, 2 equiv.), oxidant (0.9 mmol, 3 equiv.), solvent (3 mL), 6 h, N_2_ atmosphere. ^b^ Isolated yields. ^c^ 2.5 equiv. of (NH_4_)_2_S_2_O_8_ was used. ^d^ 3.5 equiv. of (NH_4_)_2_S_2_O_8_ was used. ^e^ 4 equiv. of (NH_4_)_2_S_2_O_8_ was used.

## Data Availability

The data presented in this study are available in the Supplementary Materials.

## Supplementary Materials

The following supporting information can be downloaded at https://www.mdpi.com/article/10.3390/molecules29050997/s1, Figure S1: MS spectra of compound 4a; Figure S2: MS spectra of compound 4b; Figures S3–S52: Copies of the 1H NMR and 13C NMR spectra.
