# Supplementary material for: Persulfate-Promoted Carbamoylation/Cyclization of Alkenes: Synthesis of Amide-Containing Quinazolinones
Source: Molecules. 2024 Feb 25;29(5):997. doi: 10.3390/molecules29050997 (PMC10934161; doi:10.3390/molecules29050997)
Supplement: Supplementary file 1 [file molecules-29-00997-s001.zip › molecules-2866450-supplementary.pdf]

Supporting Information

Persulfate-Promoted Carbamoylation/Cyclization of Alkenes: Synthesis of Amide-containing Quinazolinones

Jia-Jun Tang, Meng-Yang Zhao, Ying-Jun Lin, Li-Hua Yang, and Long-Yong Xie*

College of Chemistry and Bioengineering, Hunan University of Science and Engineering, Yongzhou 425100, China;

E-mail: xielongyong@huse.edu.cn

Table of Content

1. Radical Inhibition Experiment **S2**

2. ^{1}H and ^{13}C NMR spectra of products **S3**

1. Radical Inhibition Experiment

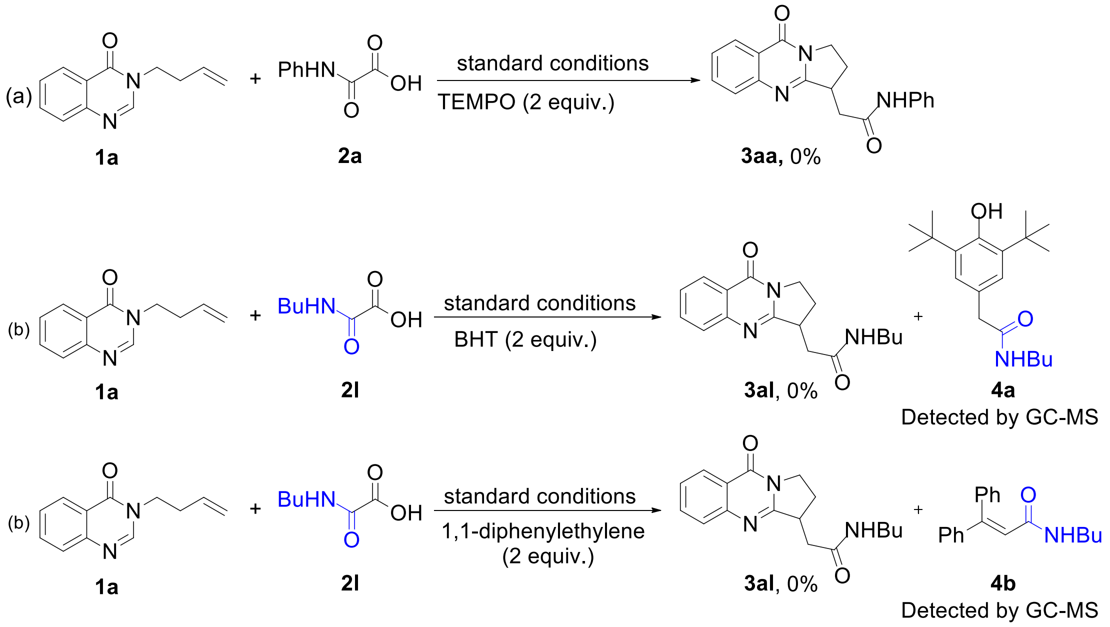

An oven-dried reaction vessel charged with a stir bar was added 3-(but-3-en-1-yl)quinazolin-4(3*H*)-one **1a** (0.3 mmol), 2-oxo-2-(phenylamino)acetic acid **2a** (0.6 mmol, 2 eq) and $(NH_4)_2S_2O_8$ (1.05 mmol, 3.5 eq) and 2 eq. of radical inhibitor (TEMPO, BHT or 1,1-diphenylethene) in DMSO-H_2O solvent (3 mL, v/v 100:1). The reaction mixture was stirred at 100 ^{o}C under N_2 atmosphere for about 6 h, no desired product **3aa** and **3al** were obtained, and the radical adduct **4a** and **4b** were detected by GC-MS (for compound **4a**, MS: M^+ calcd for $C_{20}H_{33}NO_2$ 319.25., found 319.20). The MS spectra of compound **4a** are as following (Figure S1).

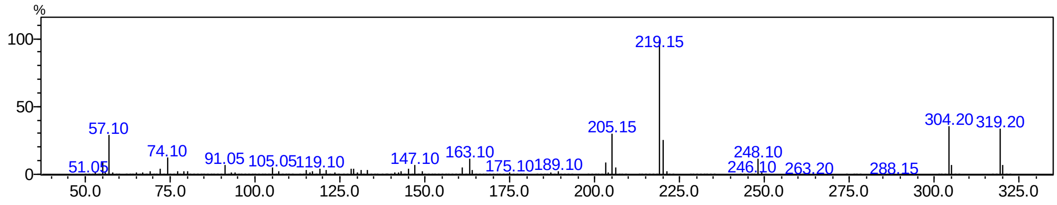

Figure S1. MS spectra of compound **4a**

For compound **4b**, MS: M^+ calcd for $C_{19}H_{21}NO_2$ 279.16, found 279.10, The MS spectra of compound **4b** are as following (Figure S2).

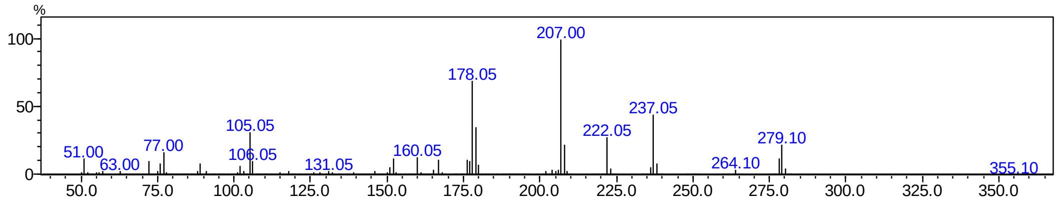

Figure S2. MS spectra of compound **4b**

2. ^{1}H and ^{13}C NMR spectra of products

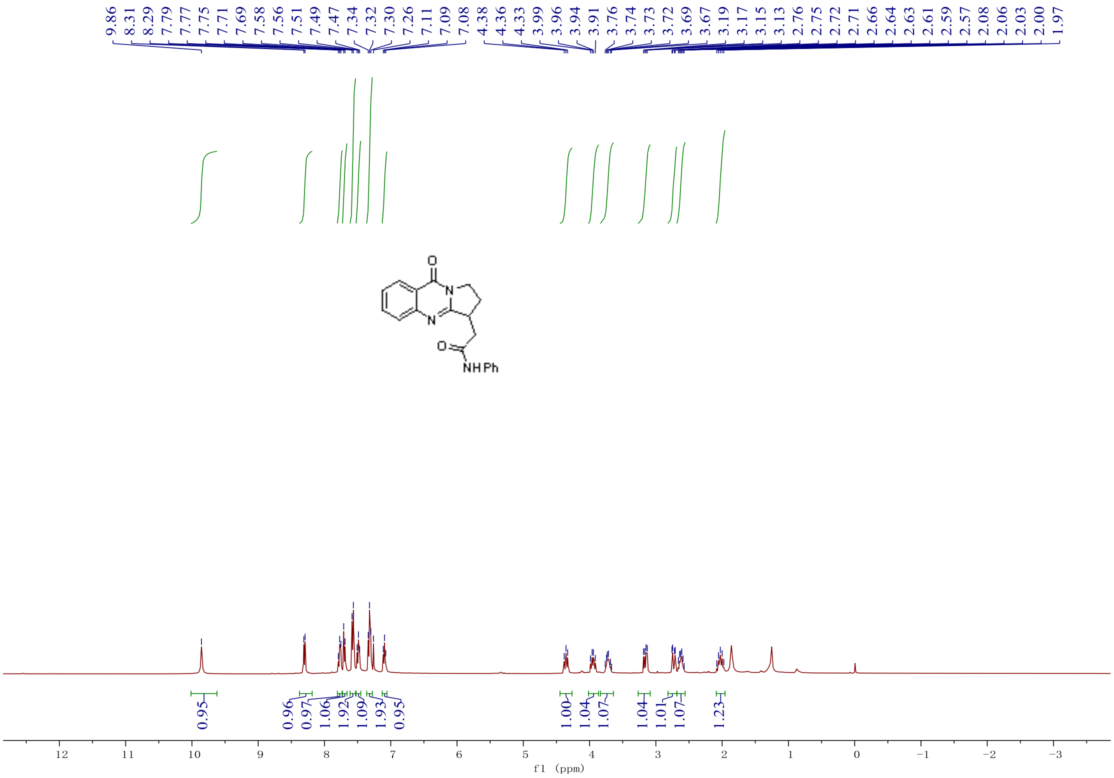

Figure S3 ^{1}H NMR spectra of **3aa**

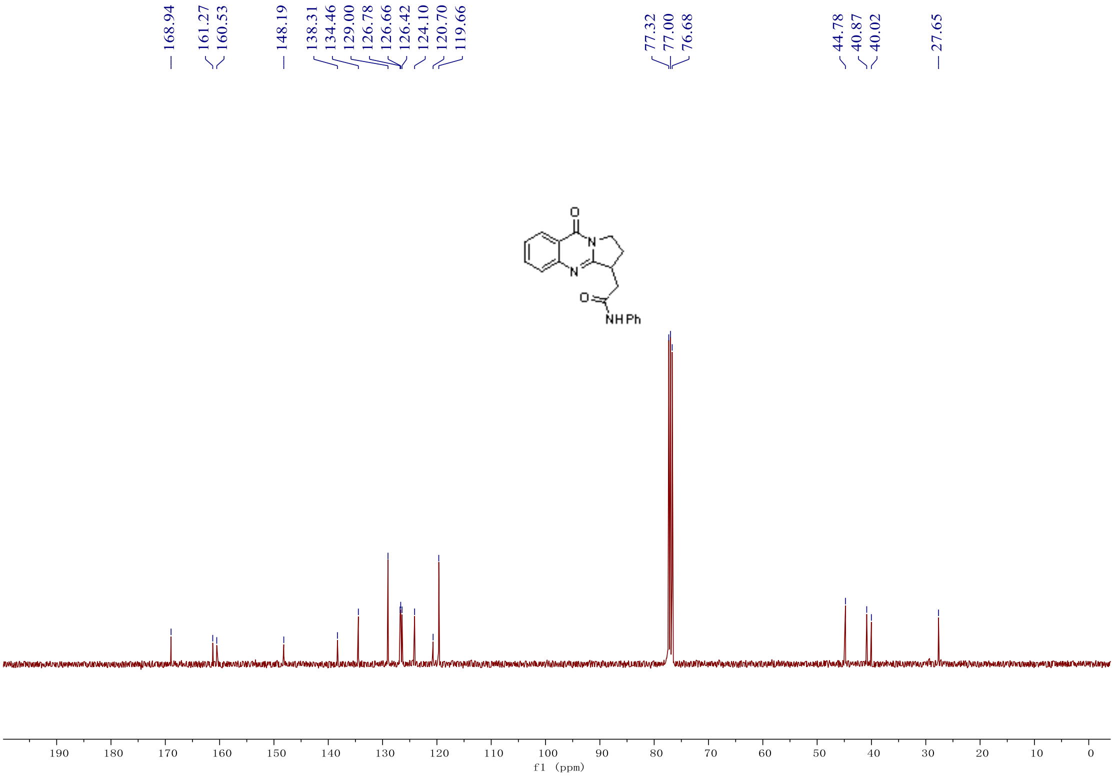

Figure S4 ^{13}C NMR spectra of **3aa**

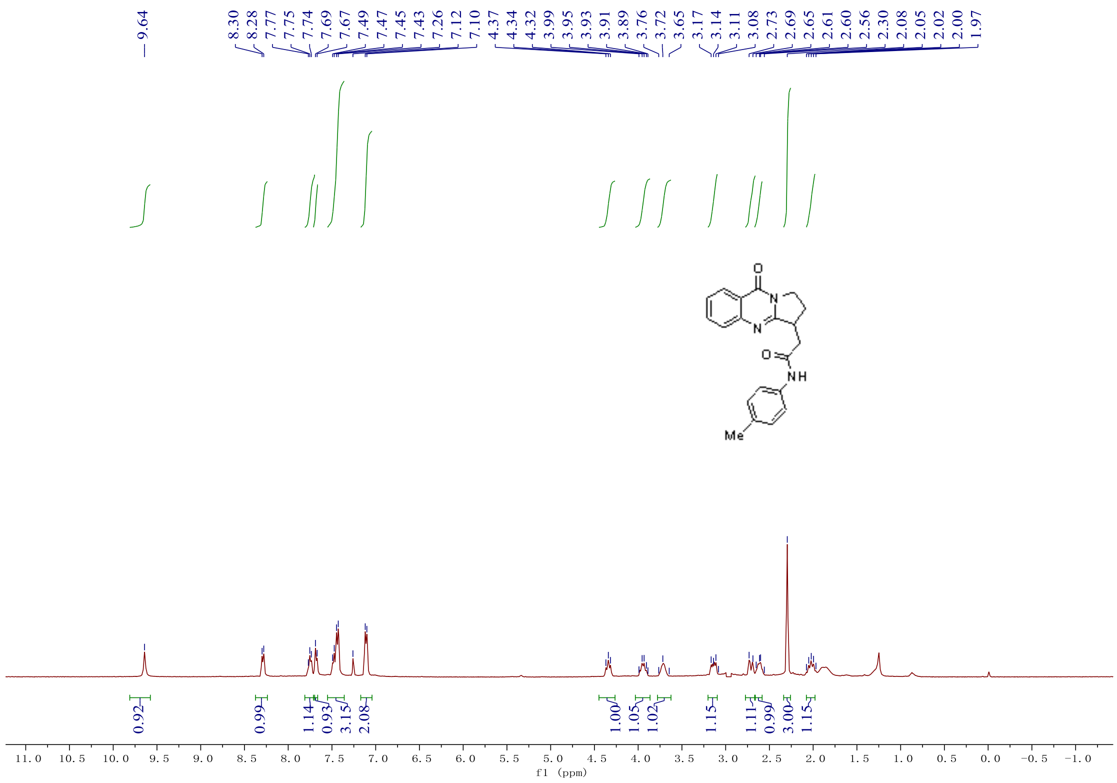

Figure S5 ^{1}H NMR spectra of **3ab**

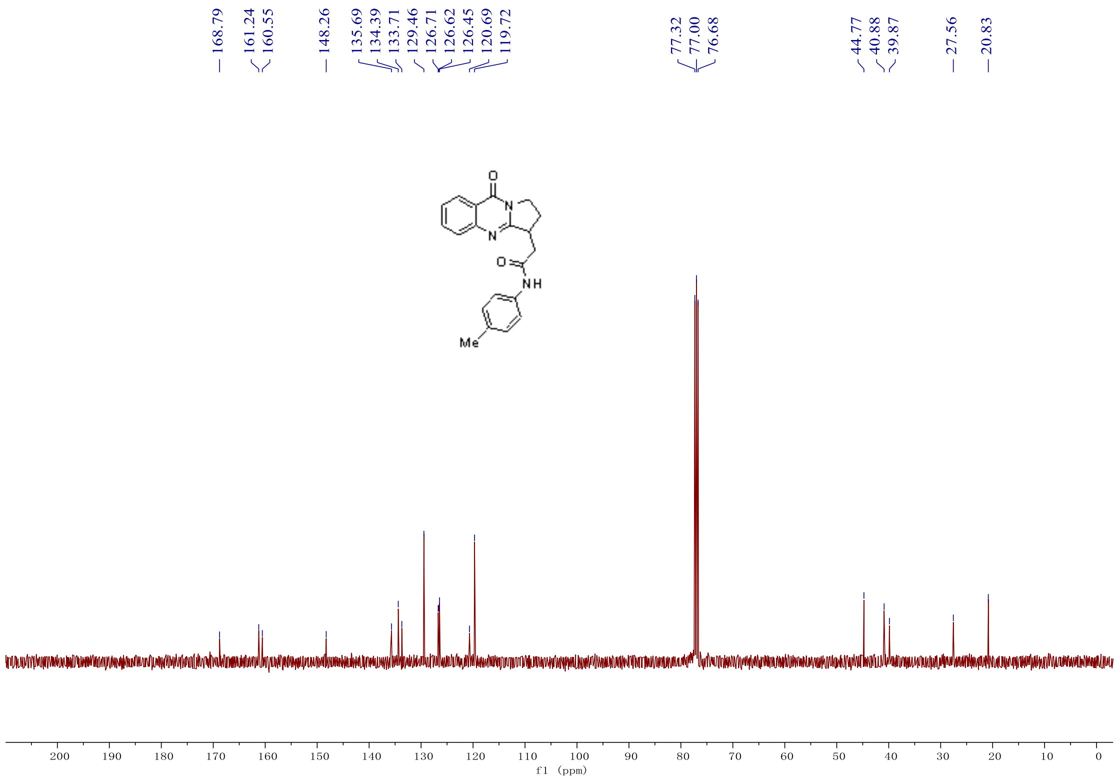

Figure S6 ^{13}C NMR spectra of **3ab**

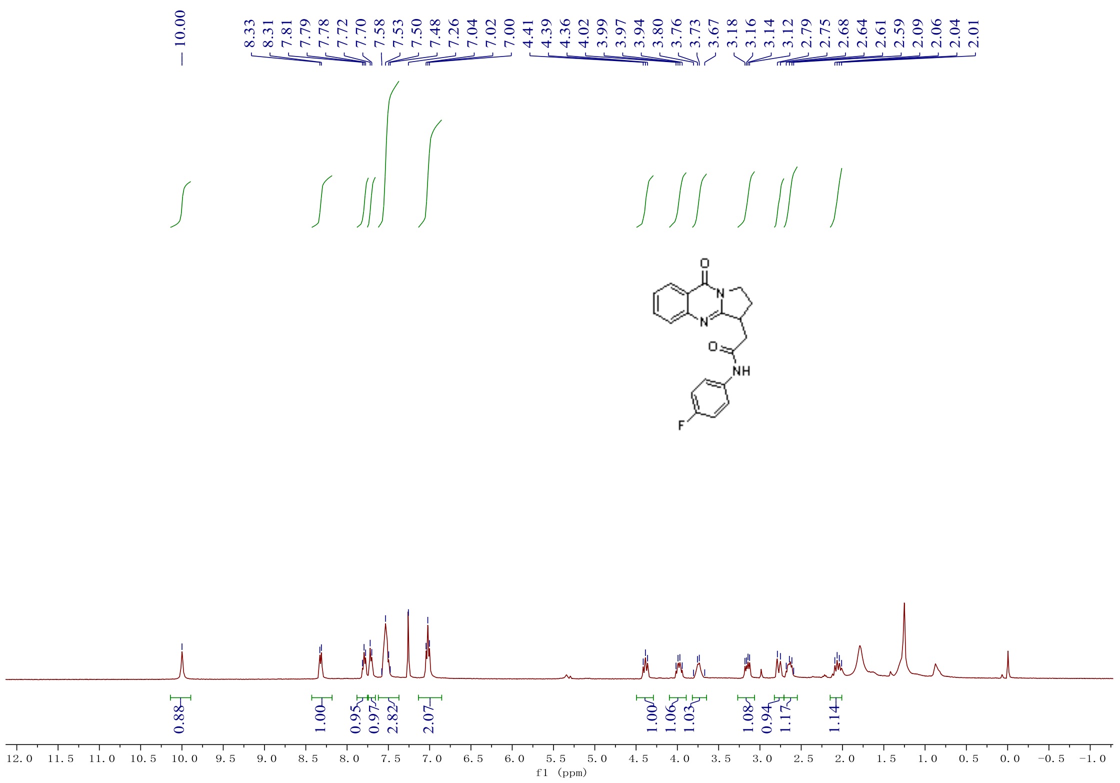

Figure S7 ^{1}H NMR spectra of **3ac**

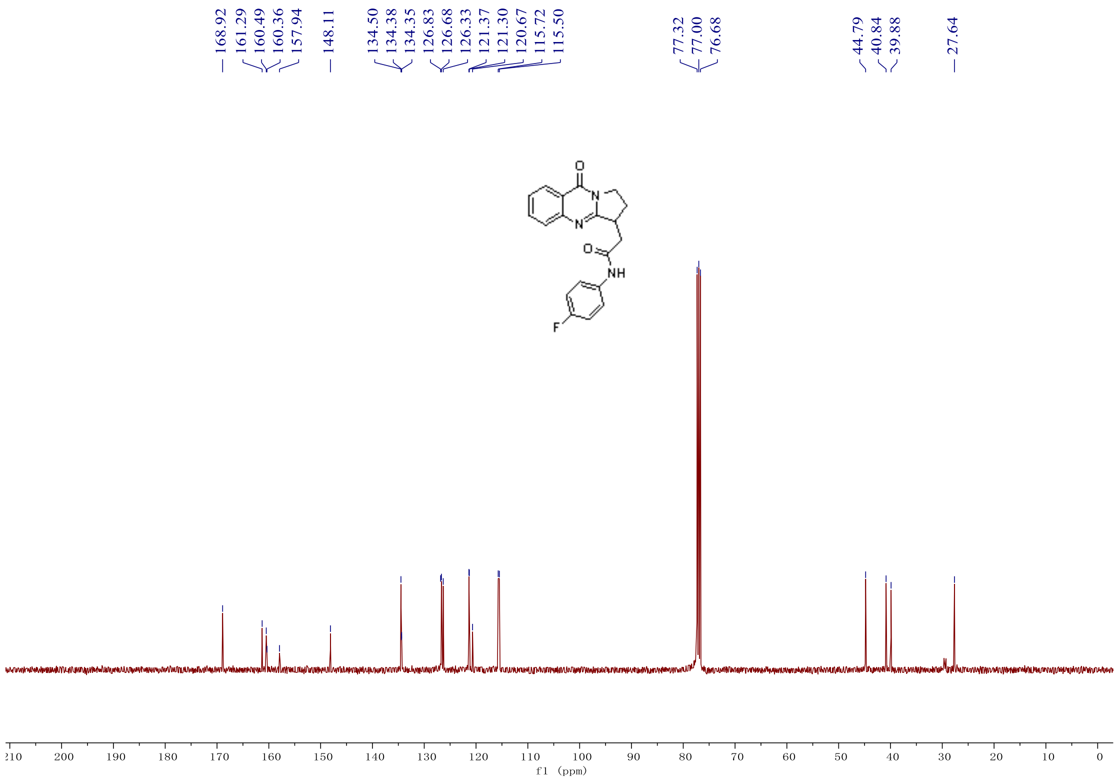

Figure S8 ^{13}C NMR spectra of **3ac**

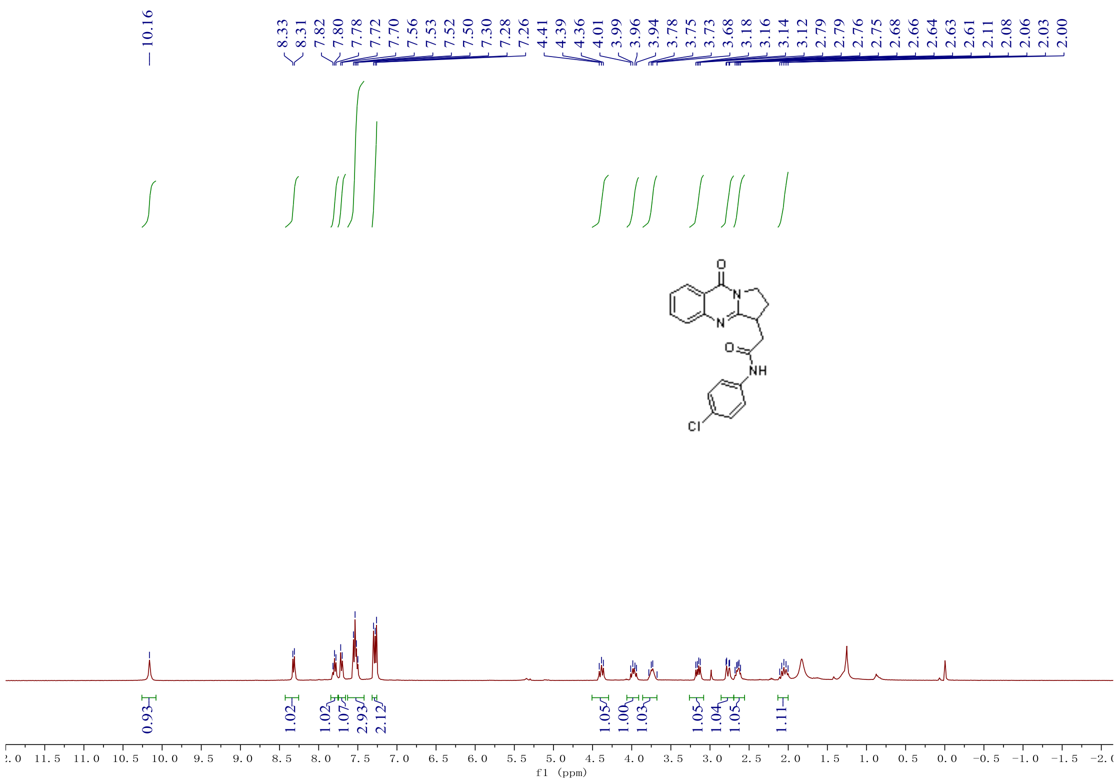

Figure S9 ^{1}H NMR spectra of **3ad**

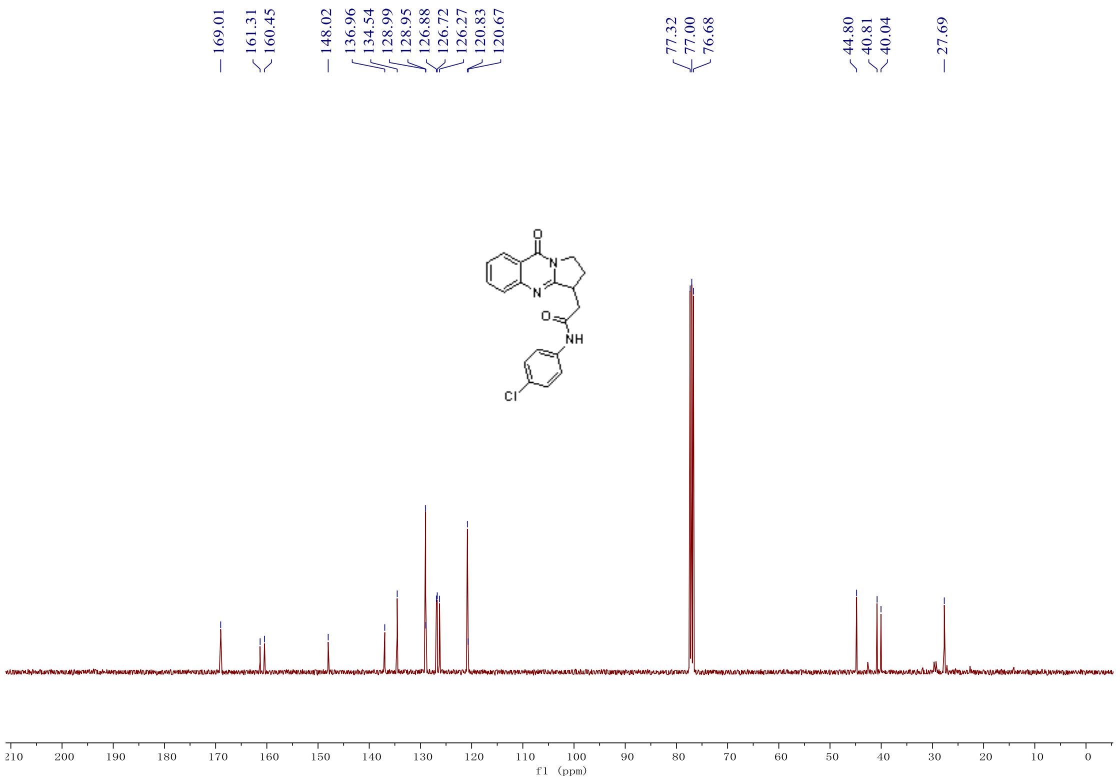

Figure S10 ^{13}C NMR spectra of **3ad**

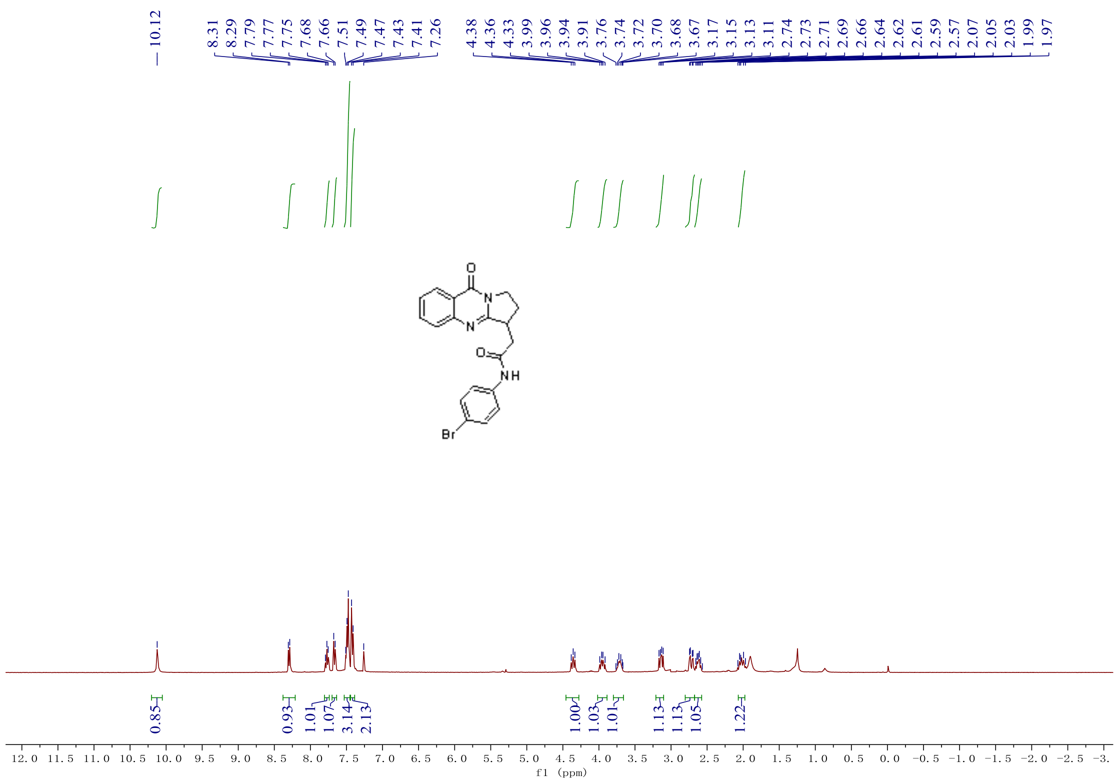

Figure S11 ^{1}H NMR spectra of **3ae**

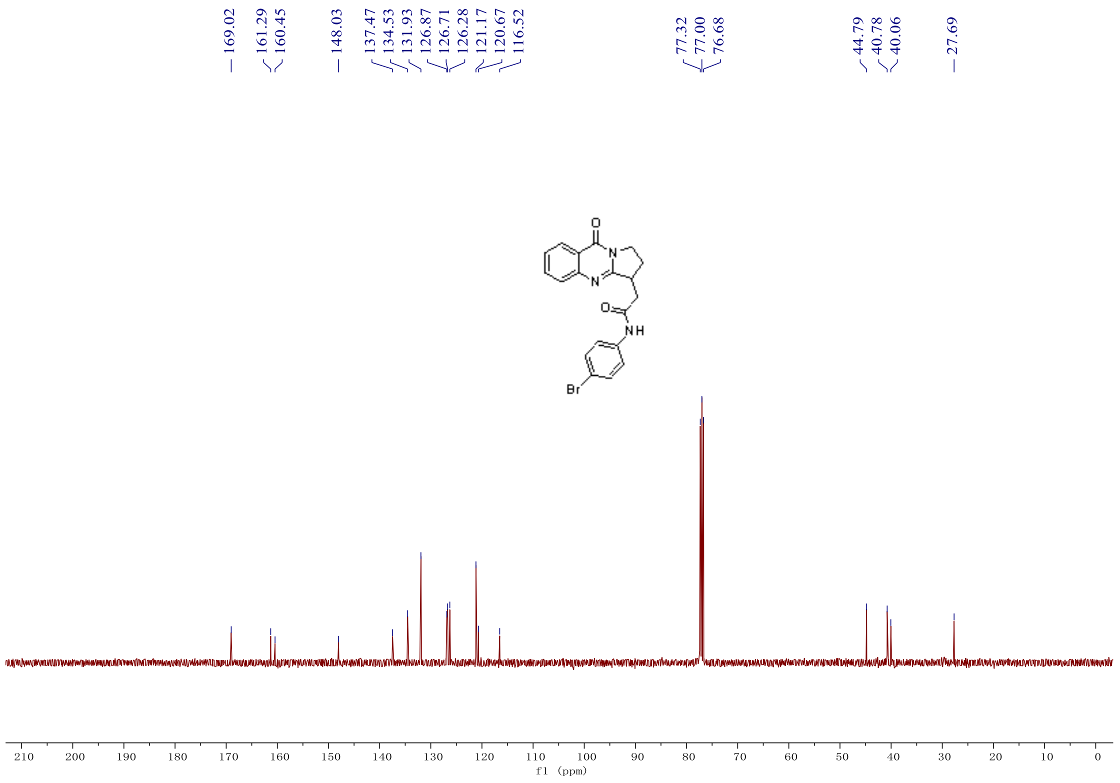

Figure S12 ^{13}C NMR spectra of **3ae**

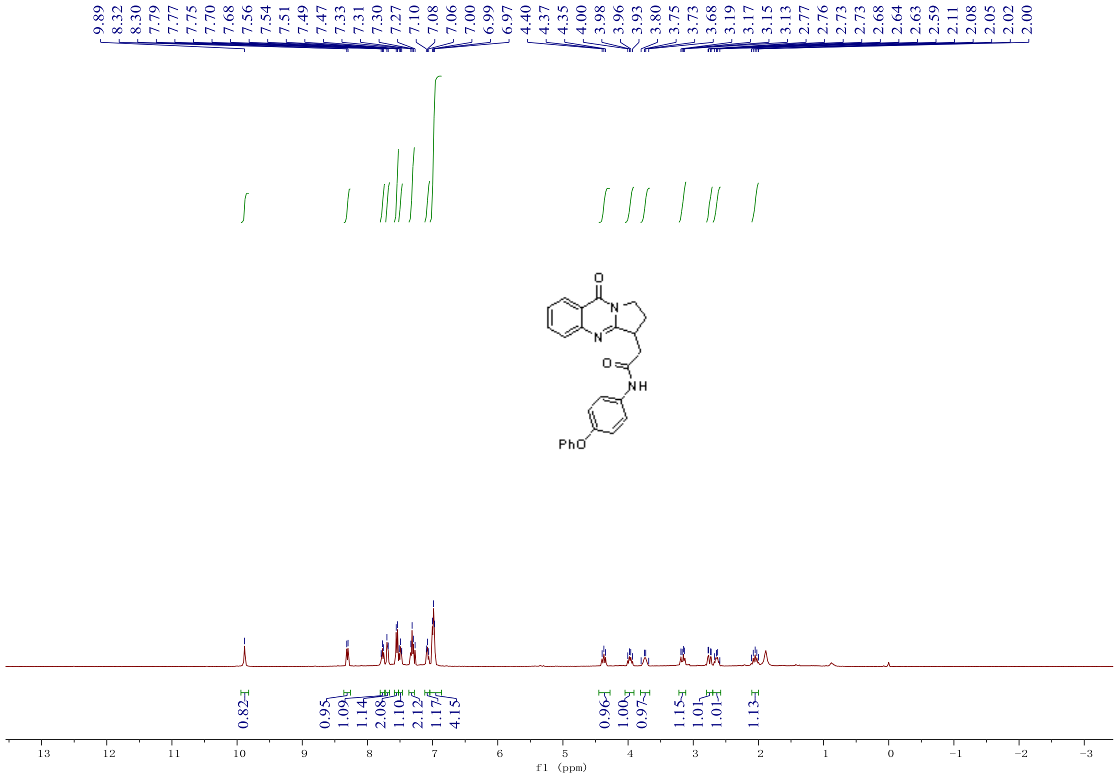

Figure S13 ^{1}H NMR spectra of **3af**

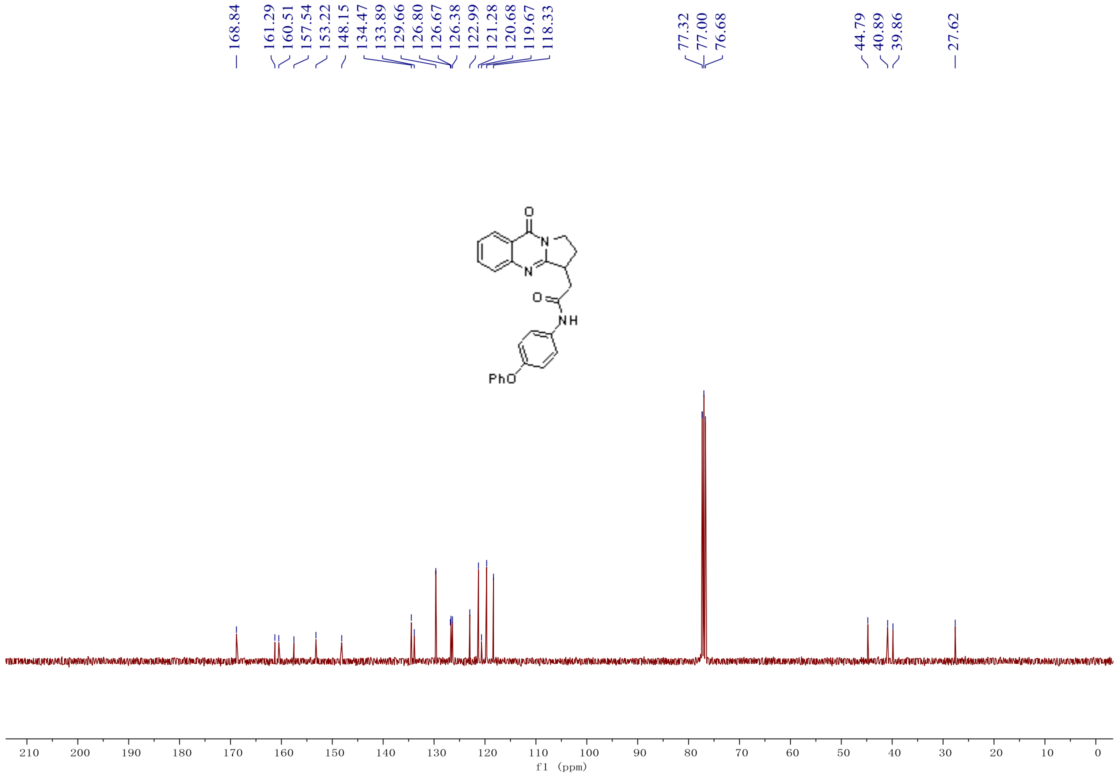

Figure S14 ^{13}C NMR spectra of **3af**

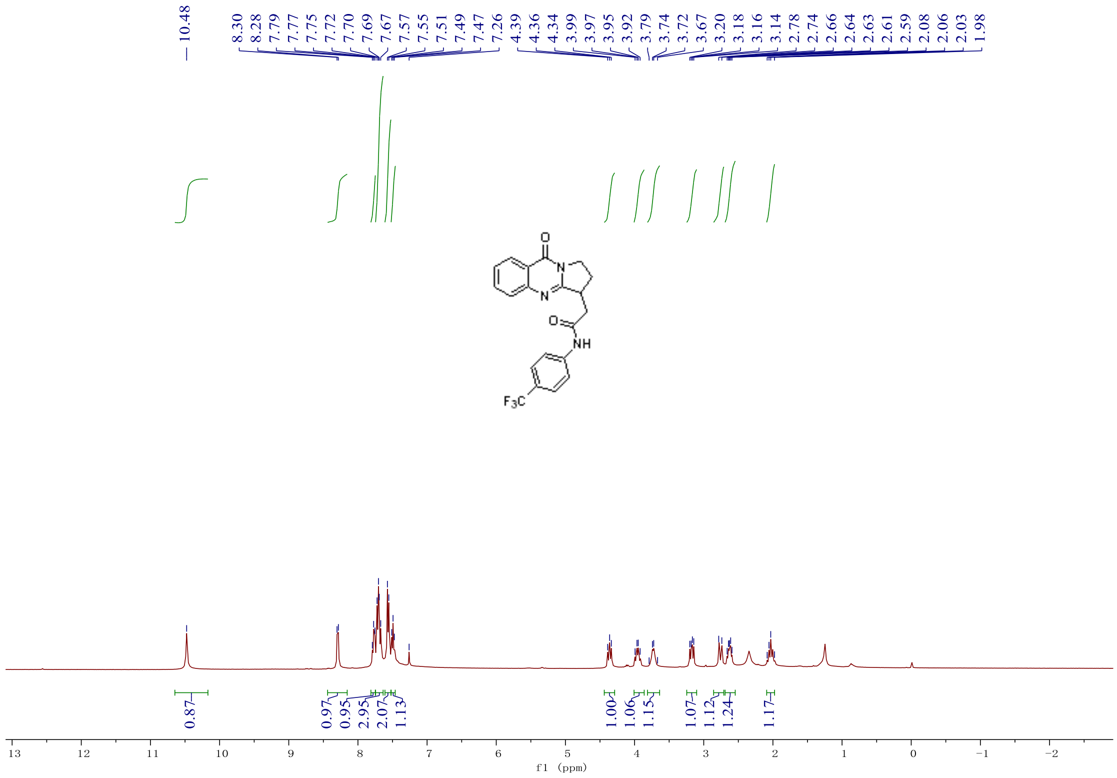

Figure S15 ^{1}H NMR spectra of **3ag**

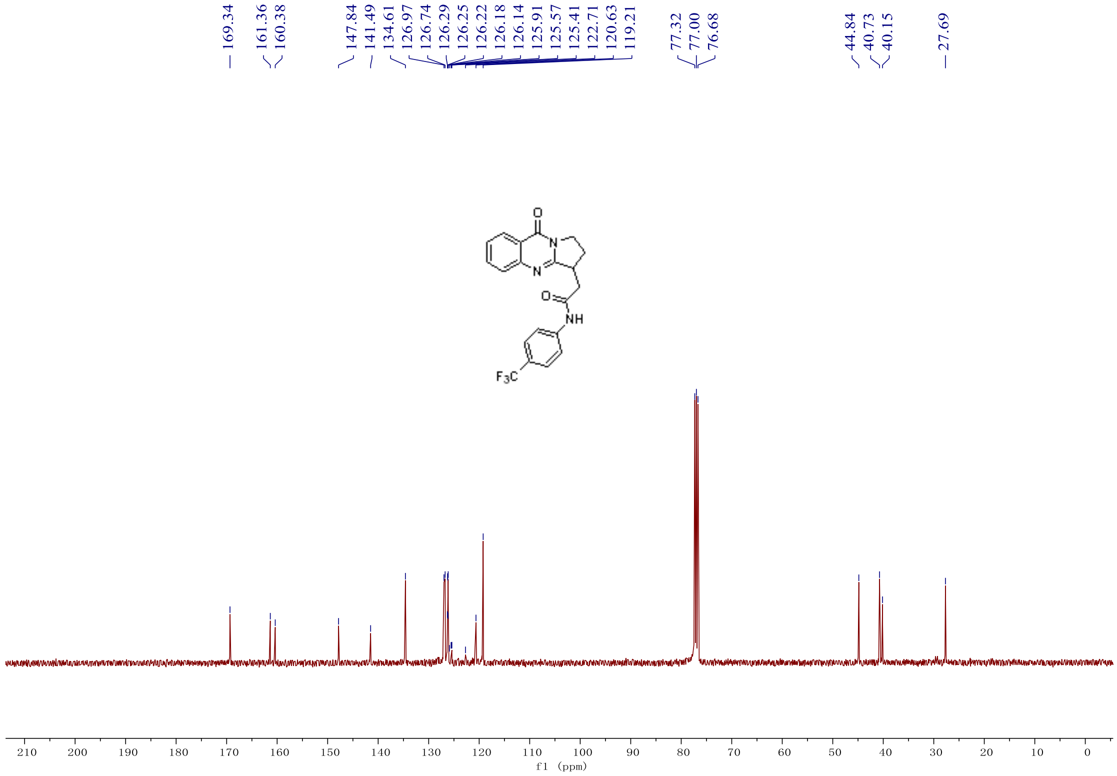

Figure S16 ^{13}C NMR spectra of **3ag**

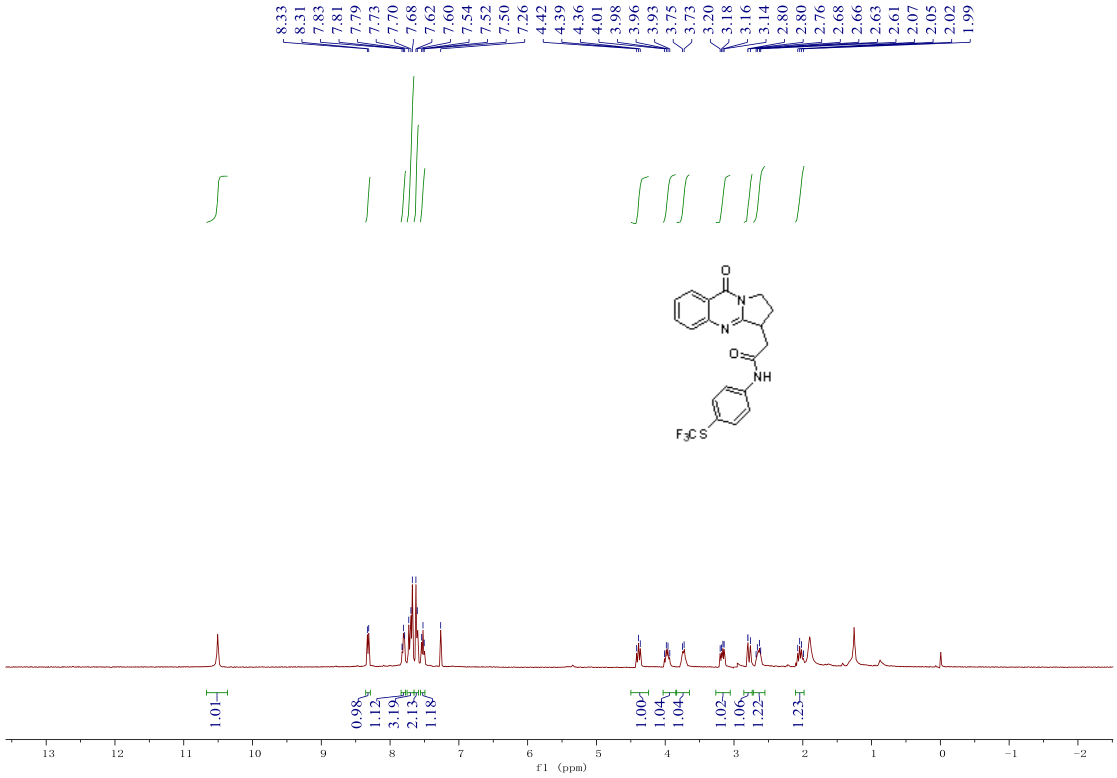

Figure S17 ^{1}H NMR spectra of **3ah**

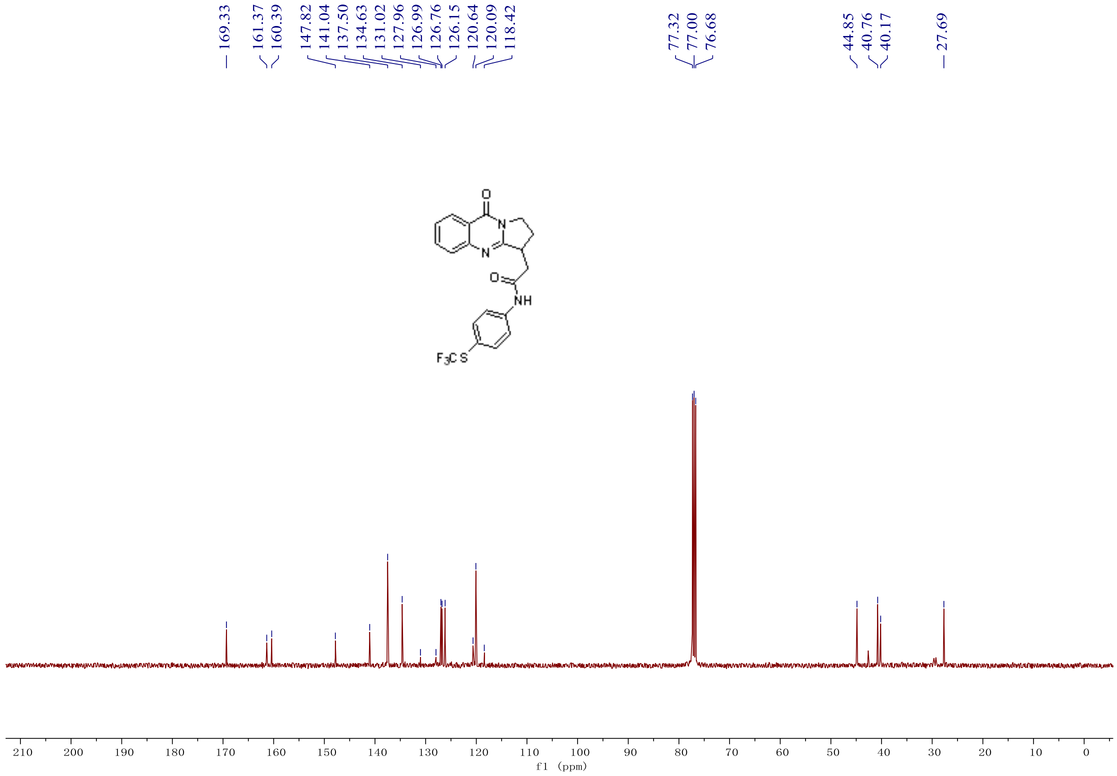

Figure S18 ^{13}C NMR spectra of **3ah**

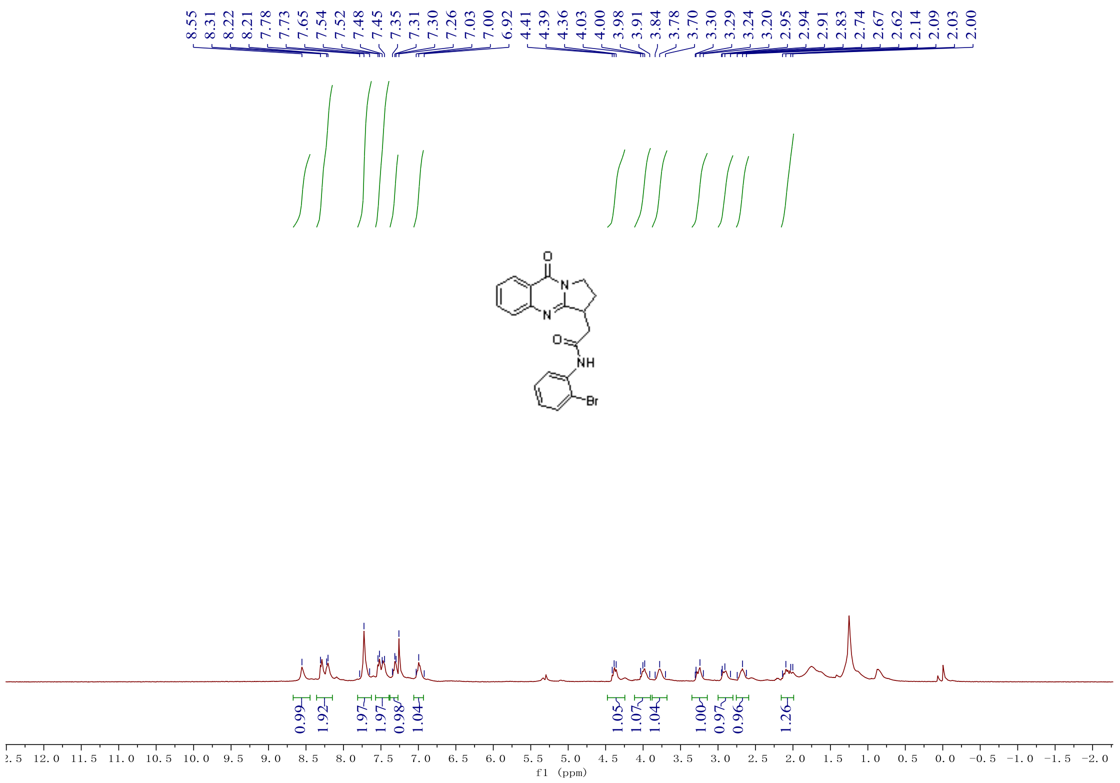

Figure S19 ^{1}H NMR spectra of **3ai**

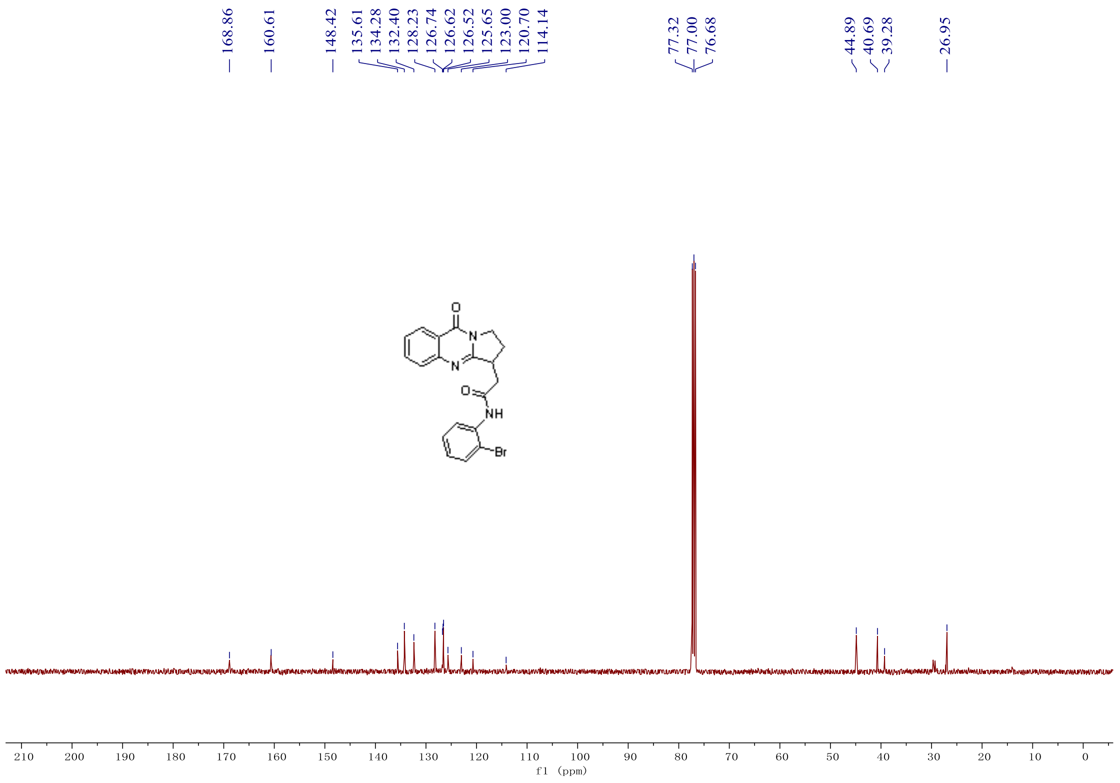

Figure S20 ^{13}C NMR spectra of **3ai**

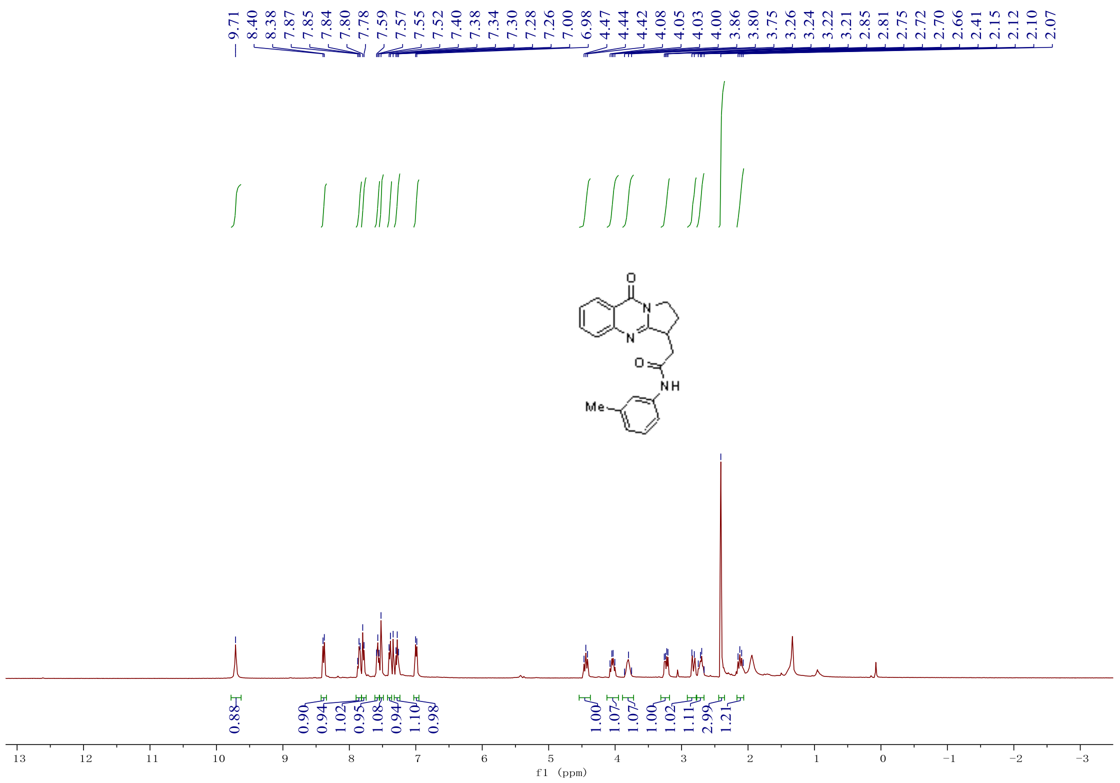

Figure S21 ^{1}H NMR spectra of **3aj**

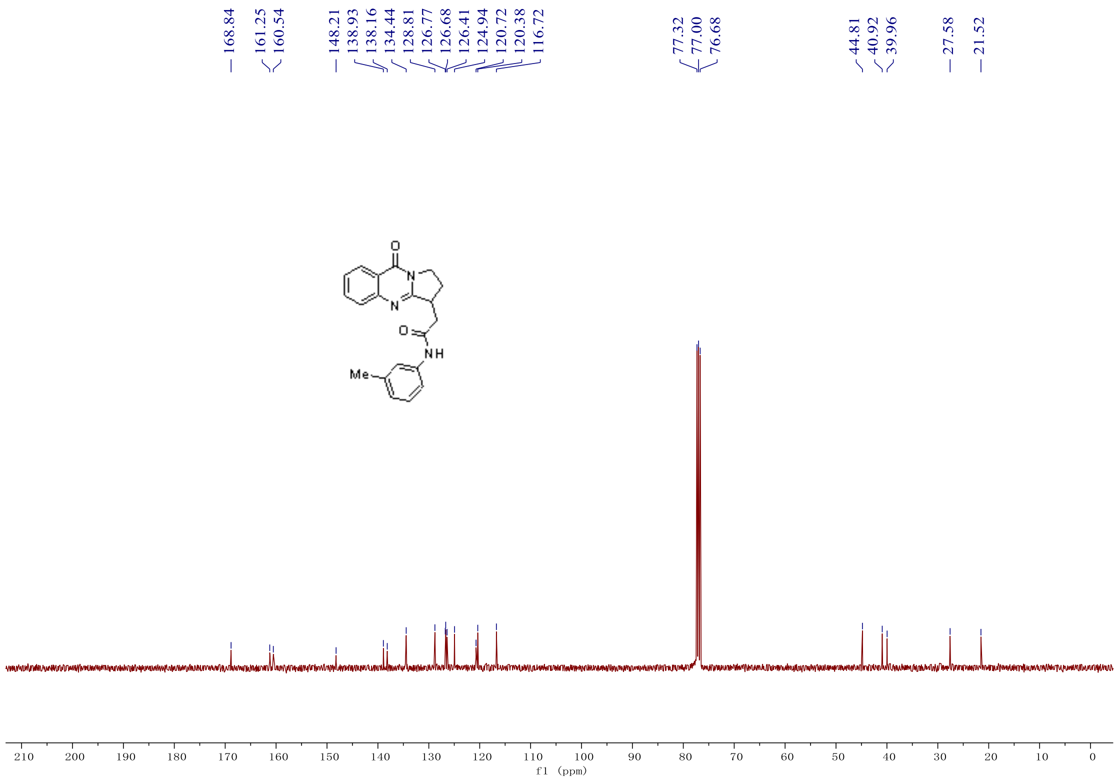

Figure S22 ^{13}C NMR spectra of **3aj**

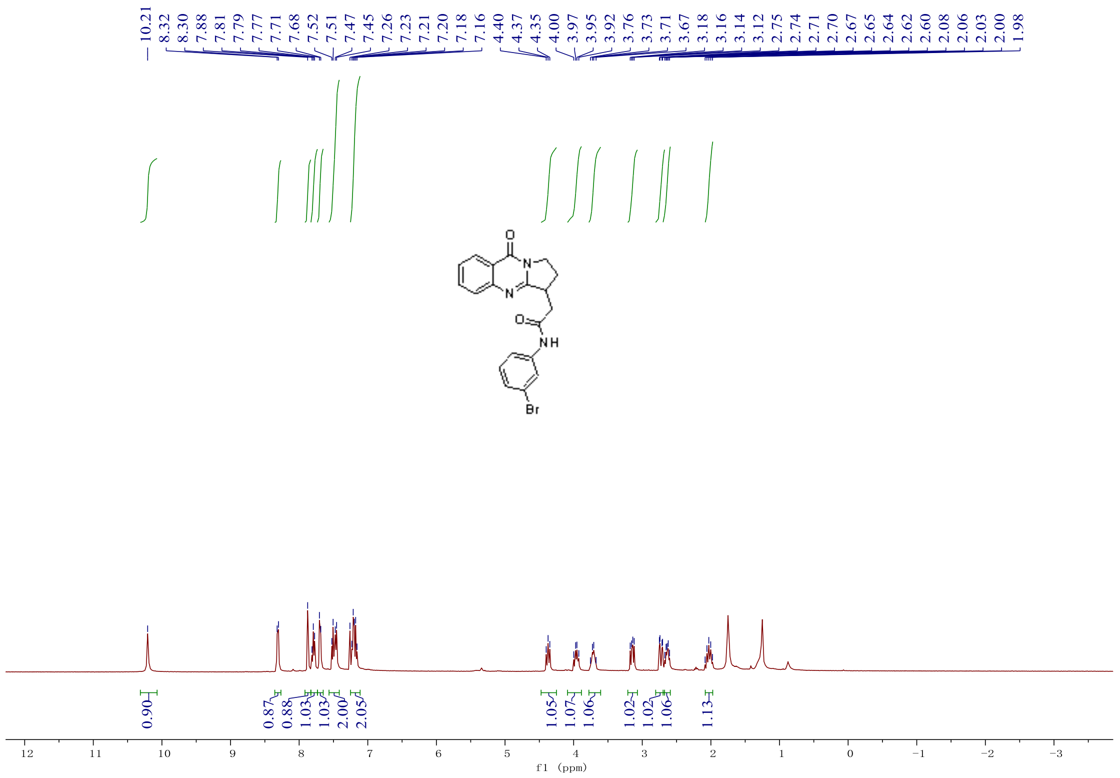

Figure S23 ^{1}H NMR spectra of **3ak**

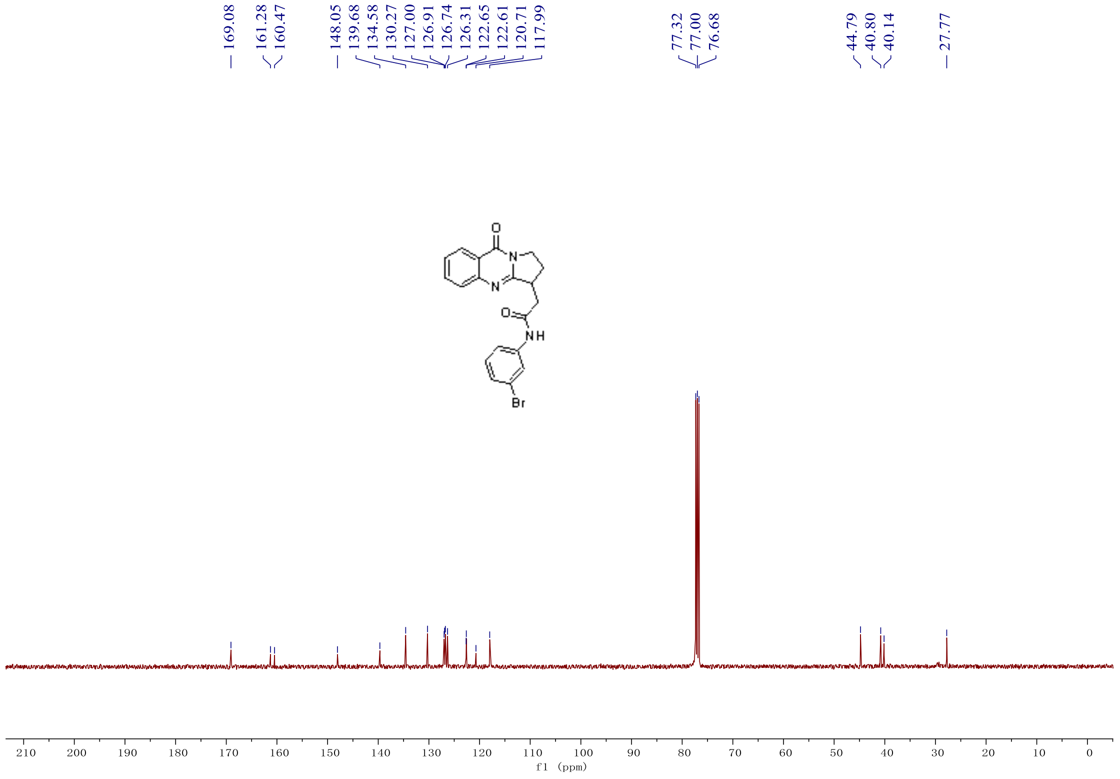

Figure S24 ^{13}C NMR spectra of **3ak**

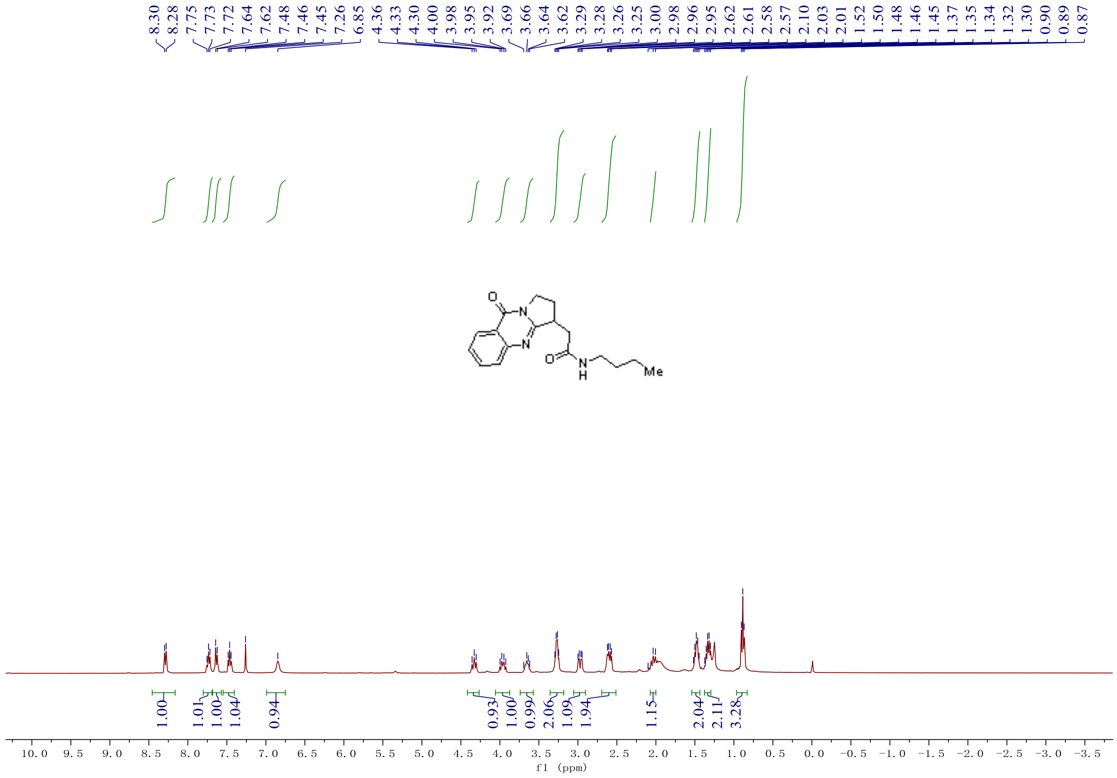

Figure S25 ^{1}H NMR spectra of **3al**

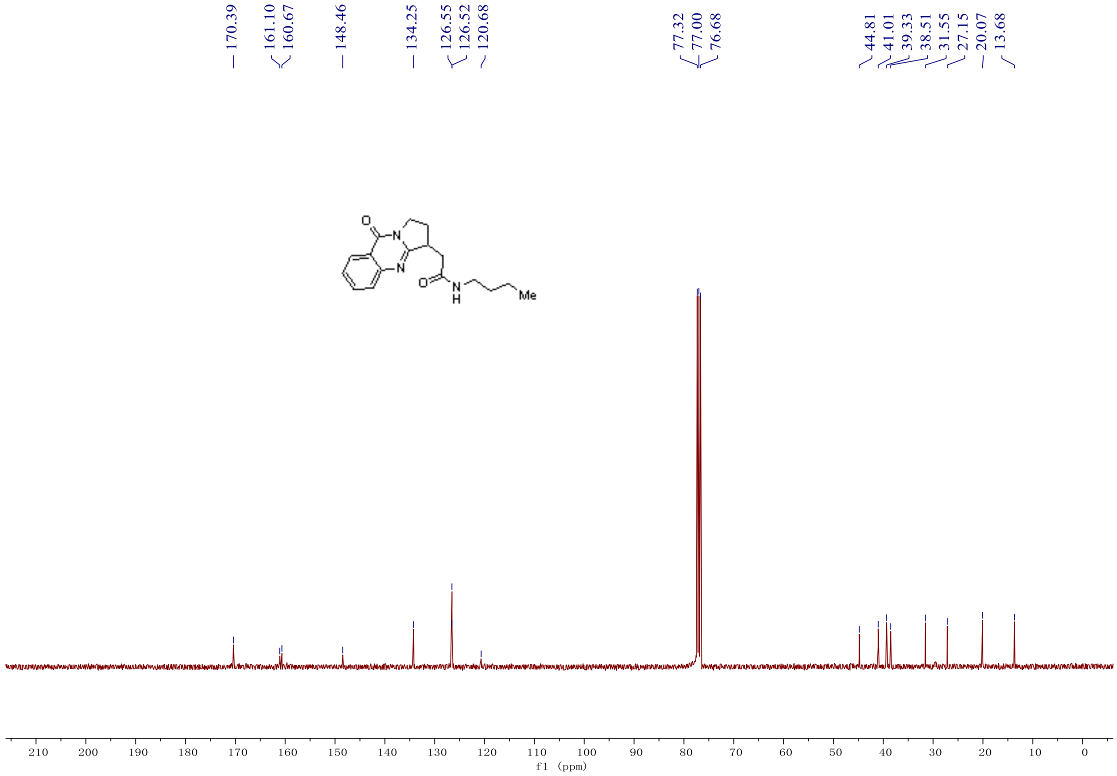

Figure S26 ^{13}C NMR spectra of **3al**

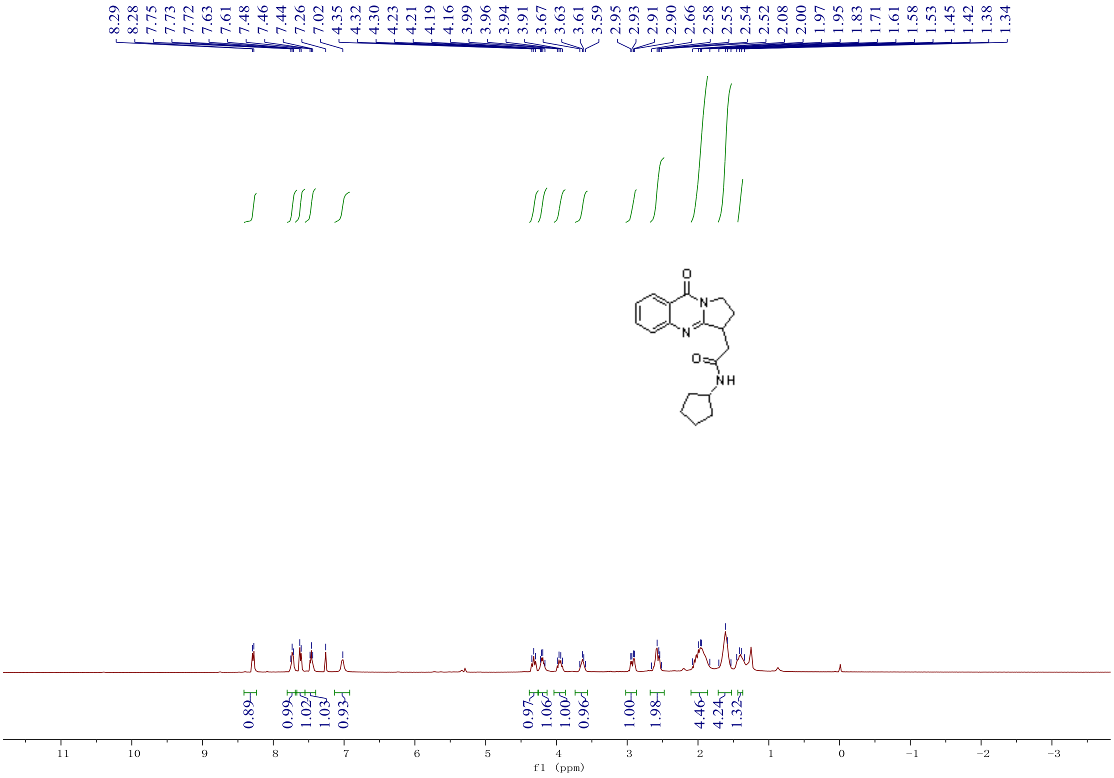

Figure S27 ^{1}H NMR spectra of **3am**

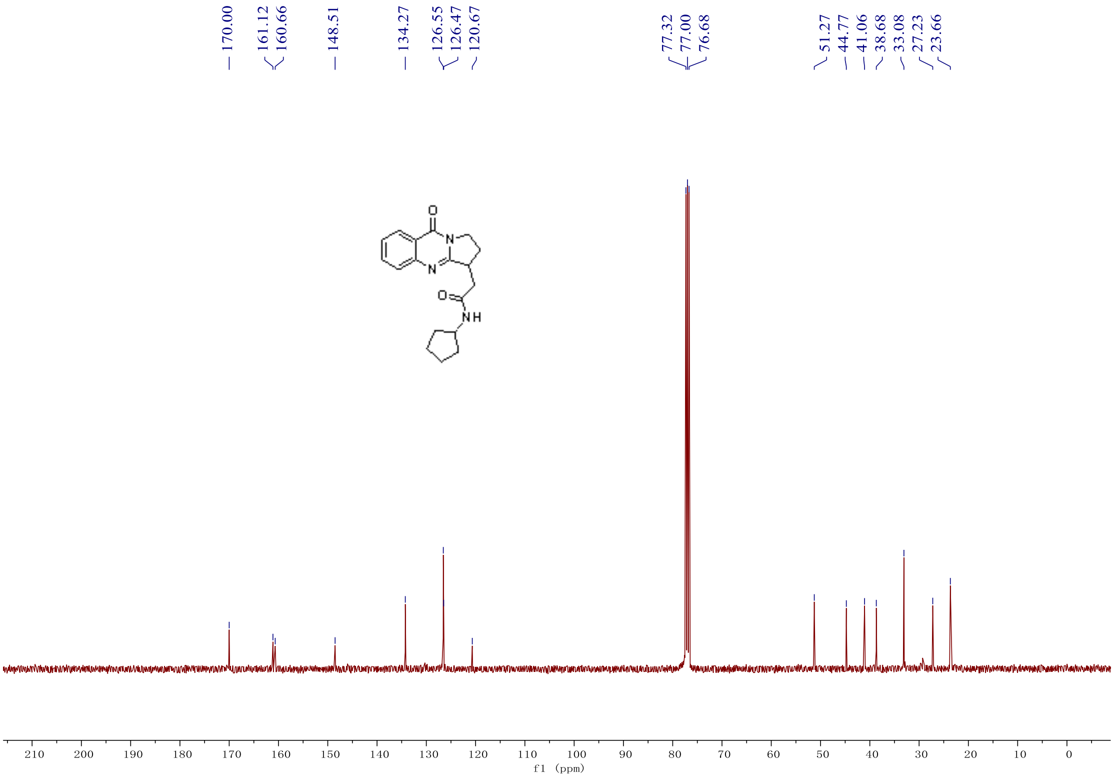

Figure S28 ^{13}C NMR spectra of **3am**

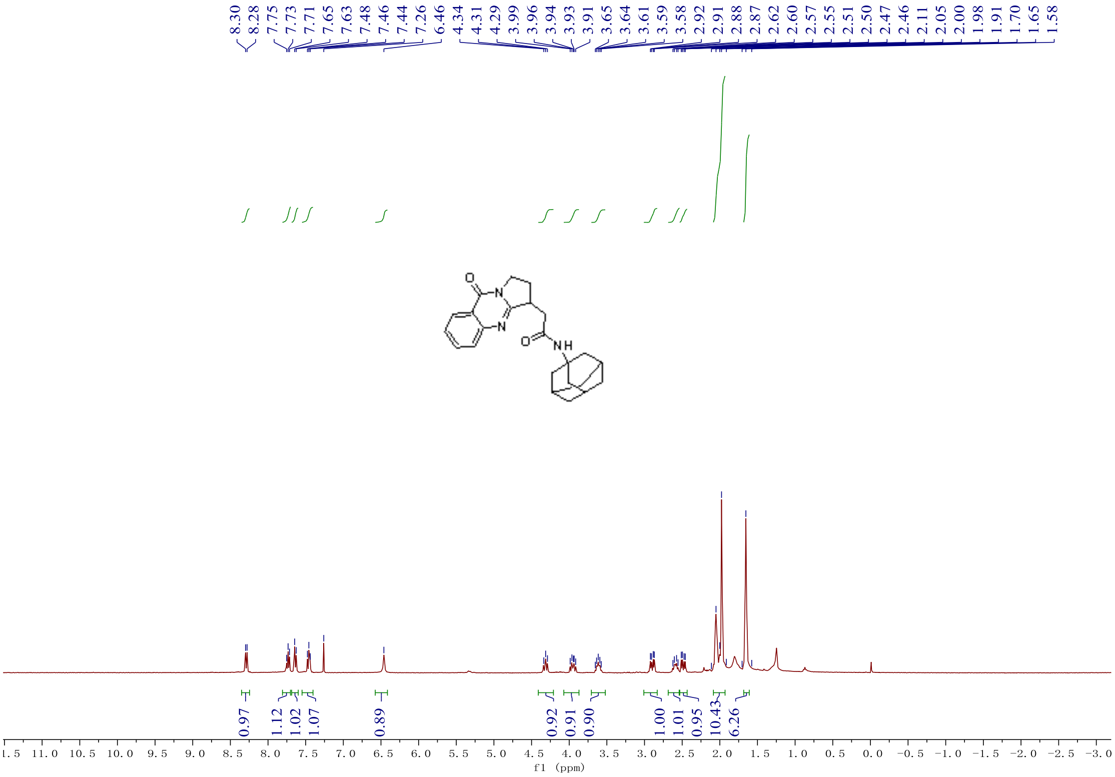

Figure S29 ^{1}H NMR spectra of **3an**

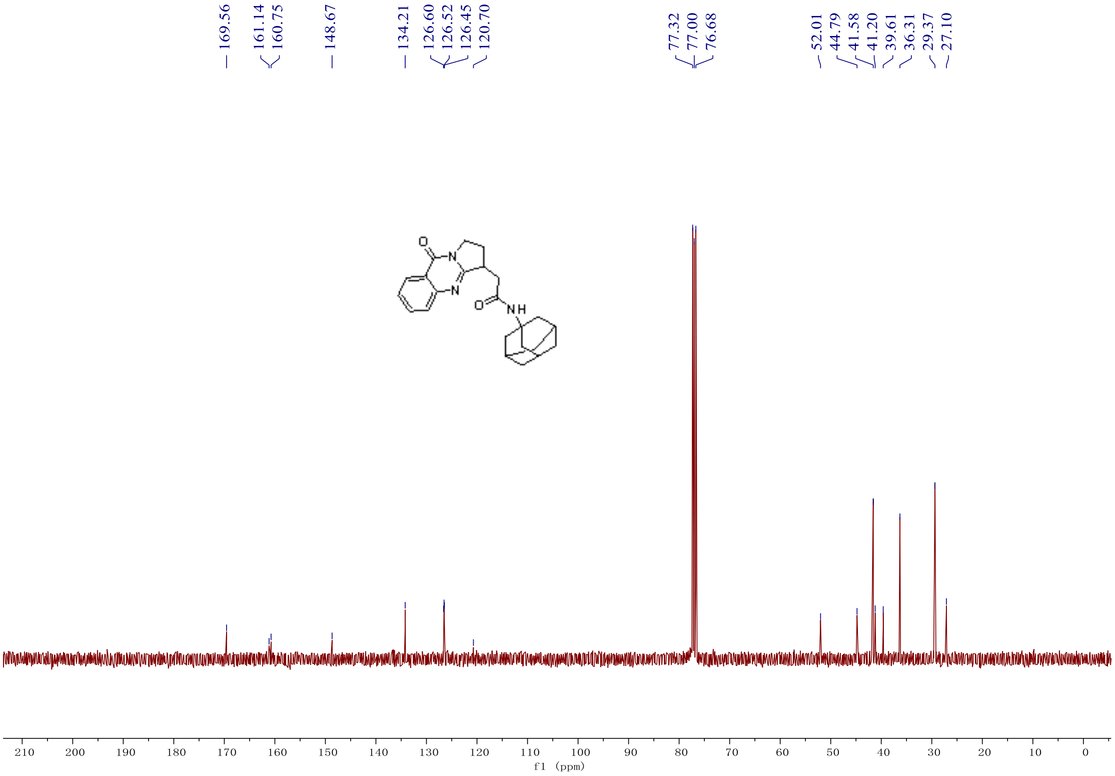

Figure S30 ^{13}C NMR spectra of **3an**

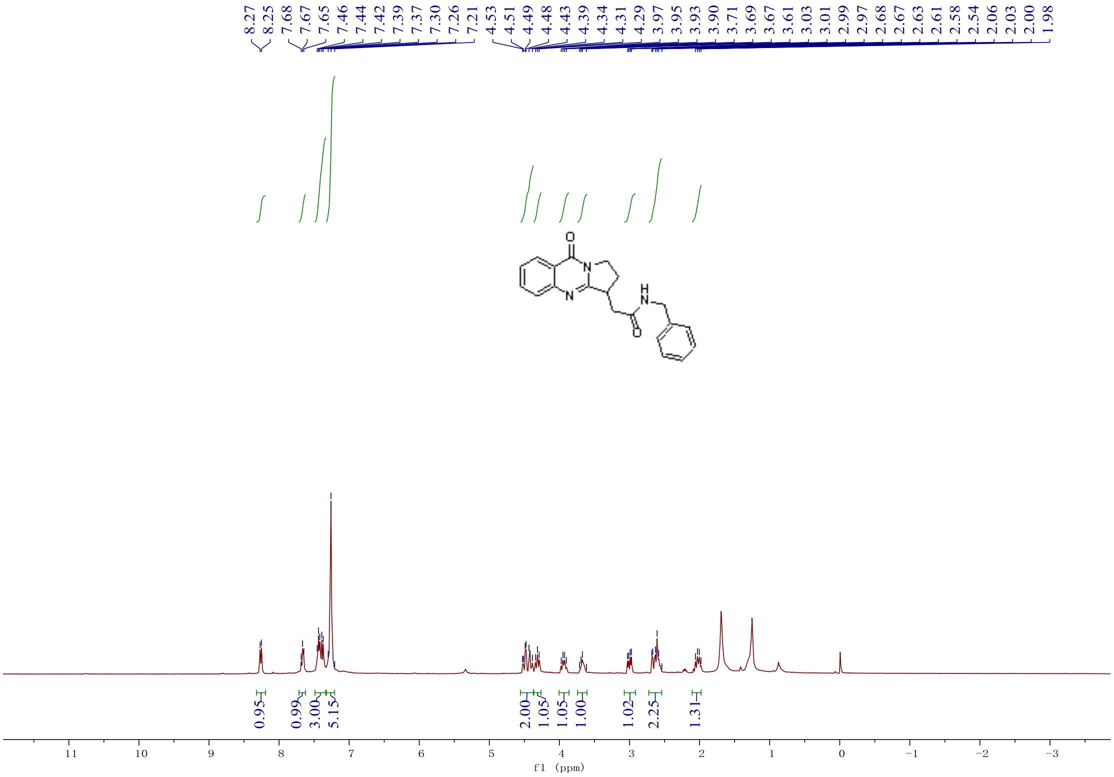

Figure S31 ^{1}H NMR spectra of **3ao**

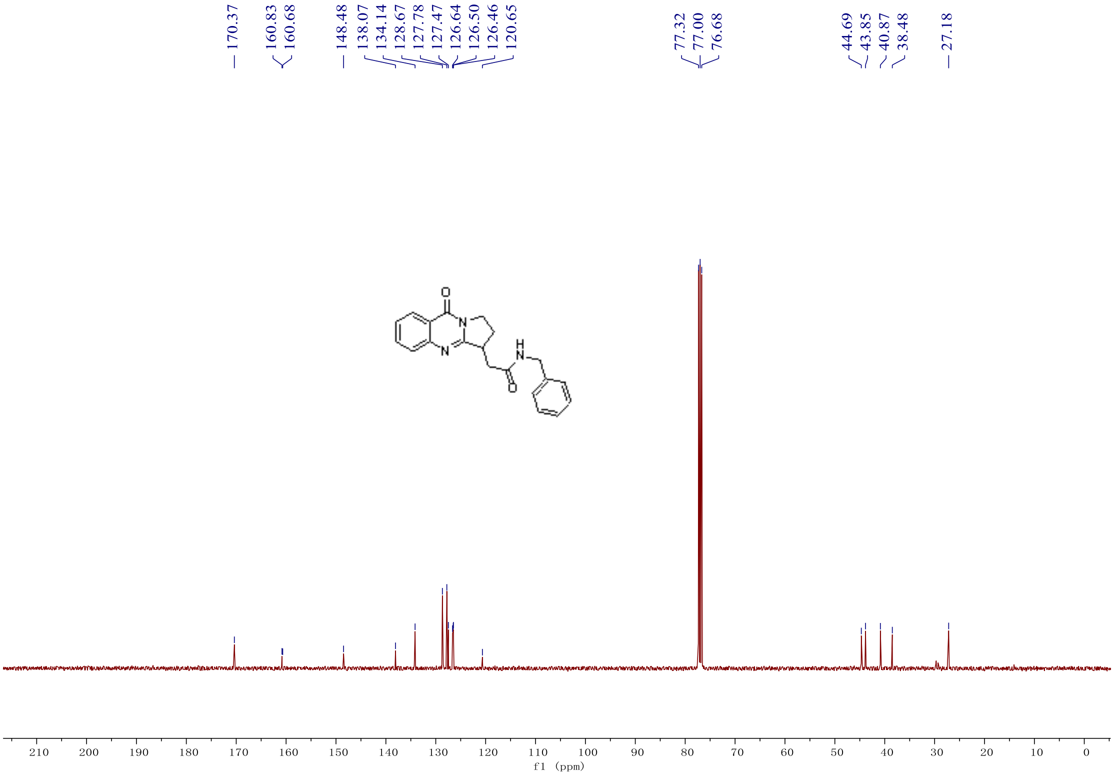

Figure S32 ^{13}C NMR spectra of **3ao**

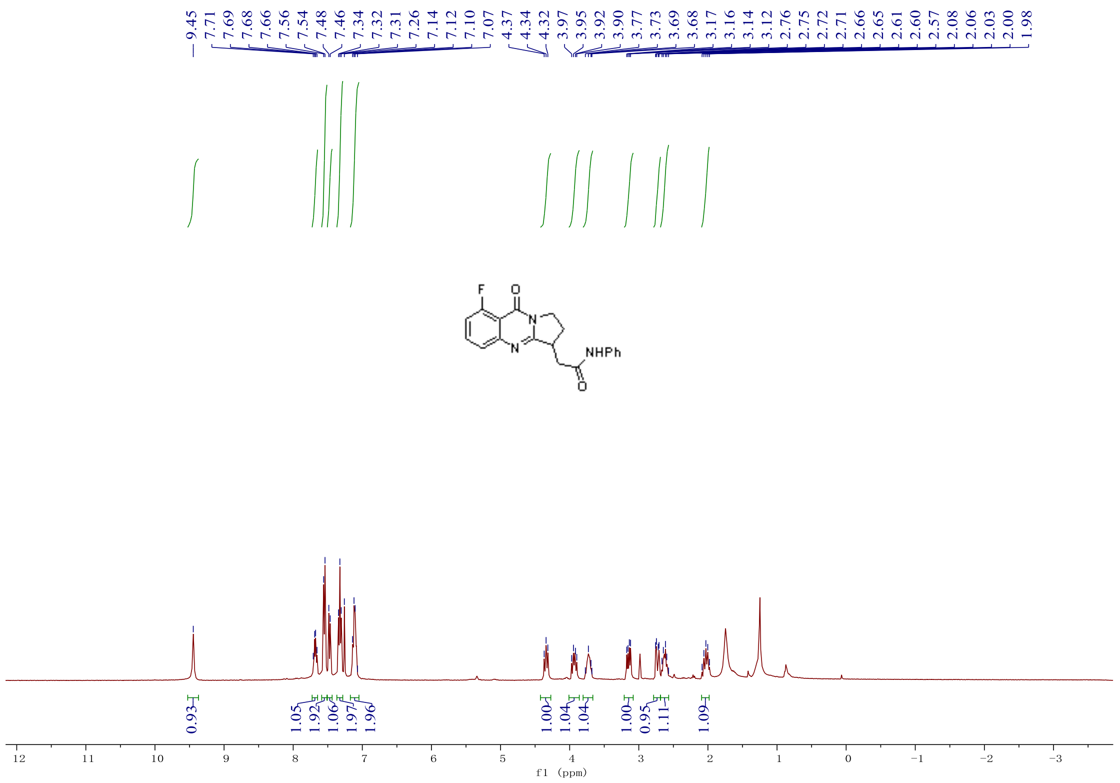

Figure S33 ^{1}H NMR spectra of **3ba**

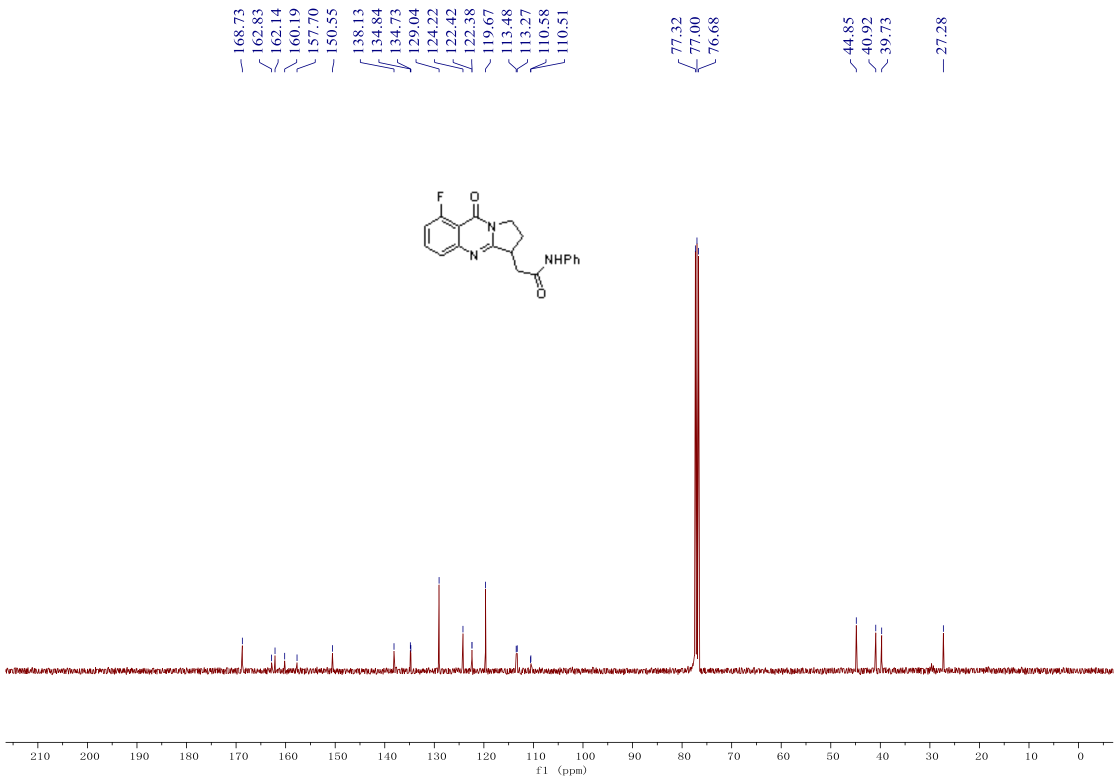

Figure S34 ^{13}C NMR spectra of **3ba**

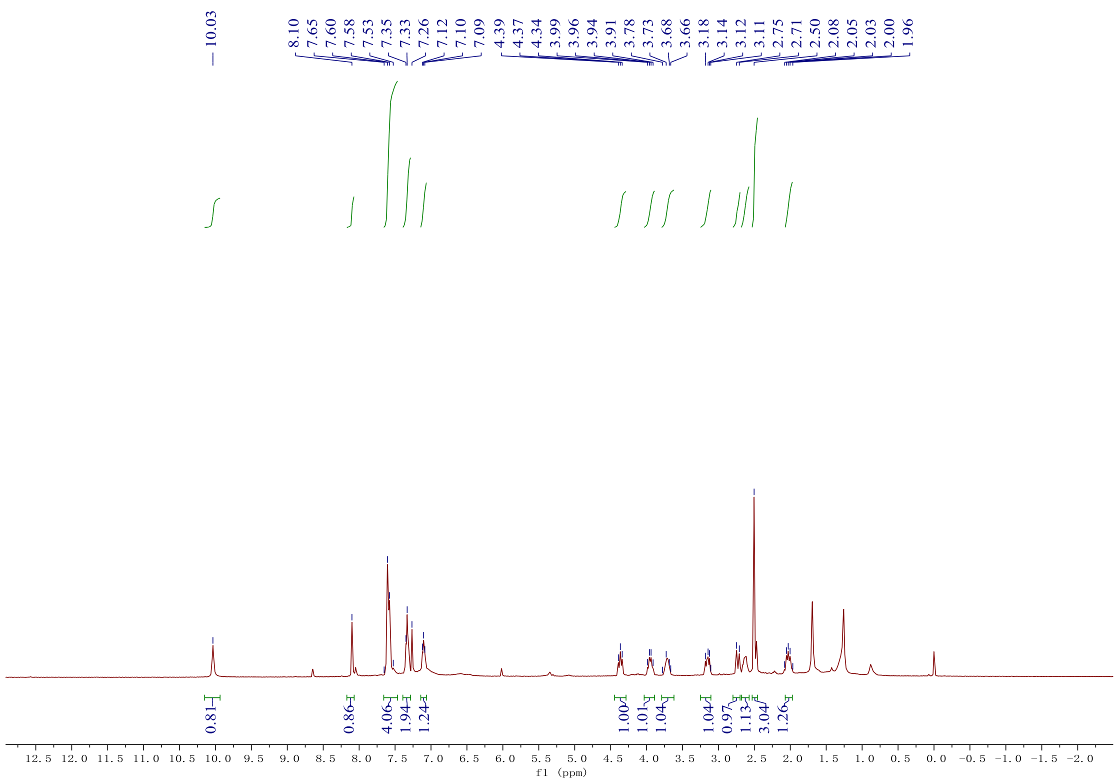

Figure S35 ^{1}H NMR spectra of **3ca**

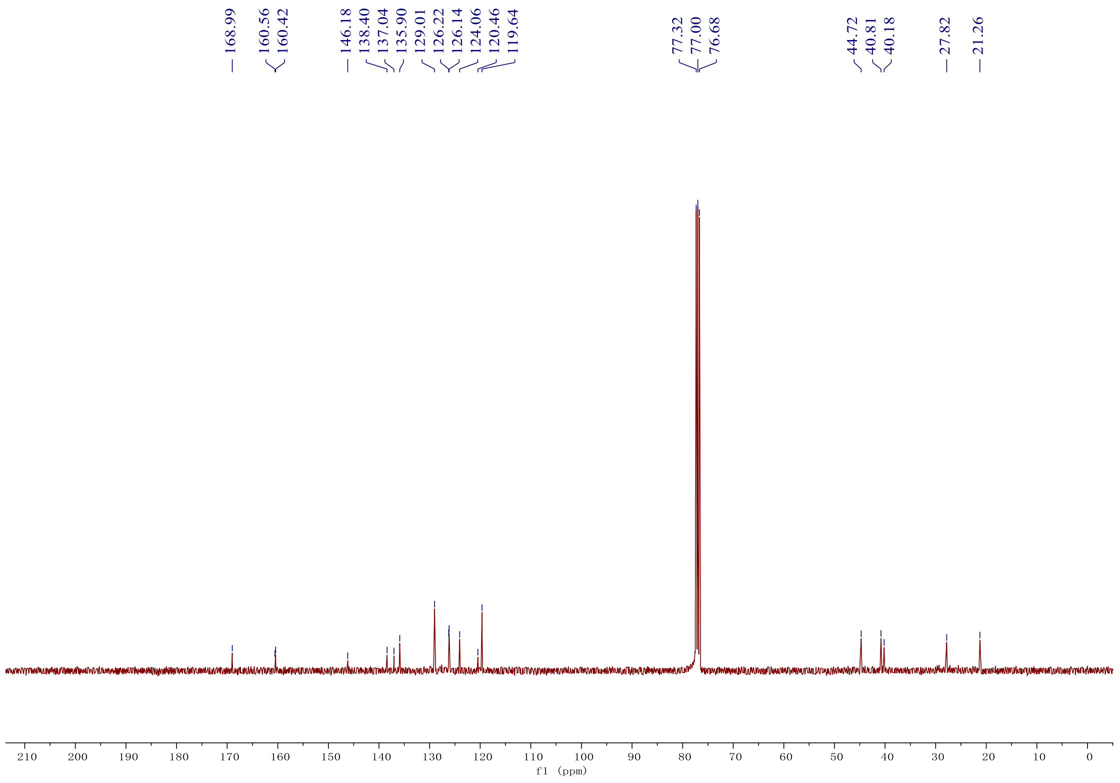

Figure S36 ^{13}C NMR spectra of **3ca**

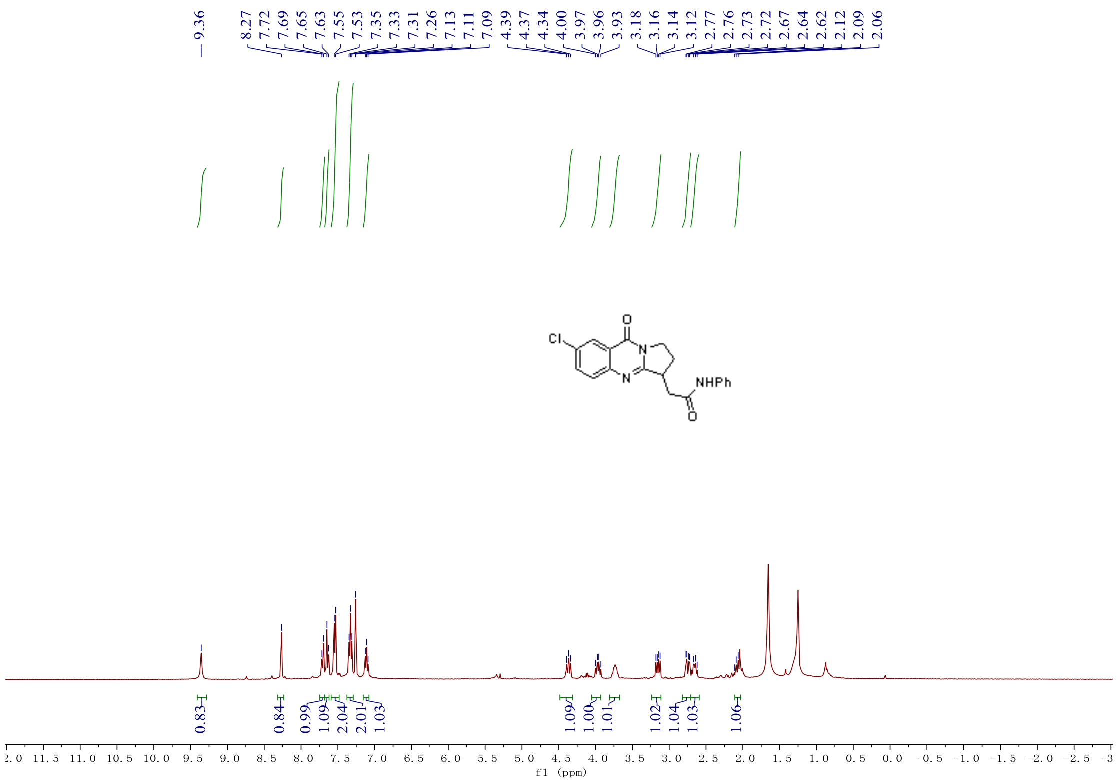

Figure S37 ^{1}H NMR spectra of **3da**

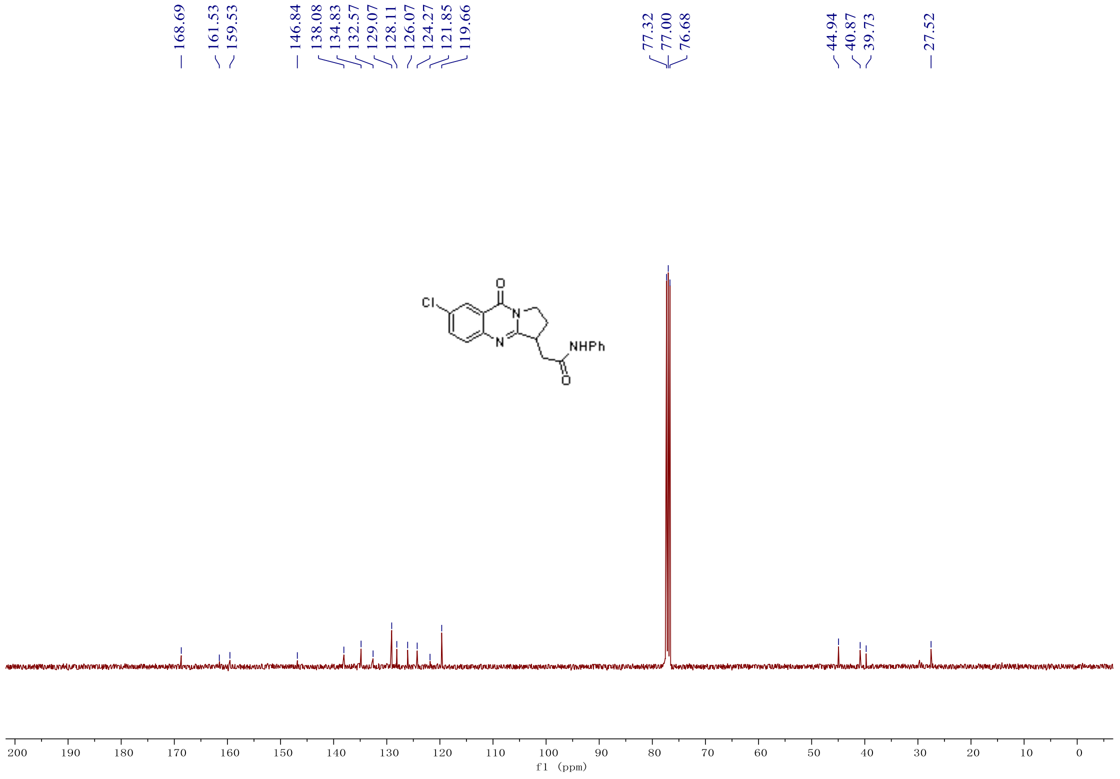

Figure S38 ^{13}C NMR spectra of **3da**

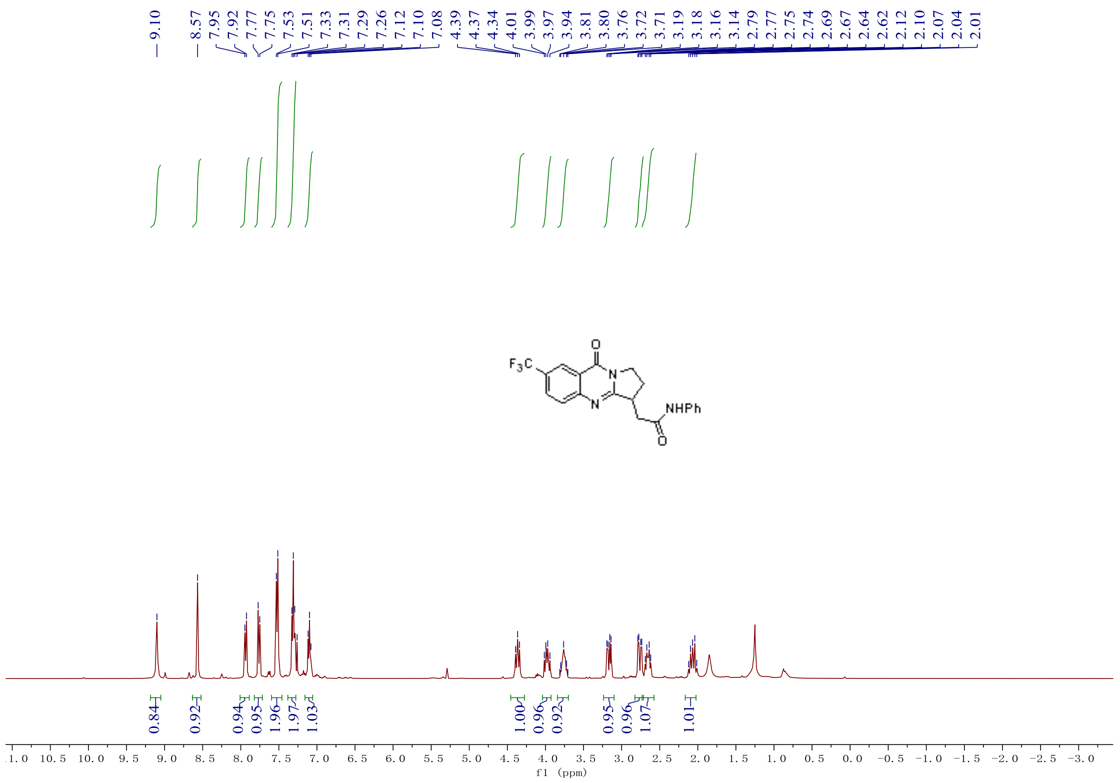

Figure S39 ^{1}H NMR spectra of **3ea**

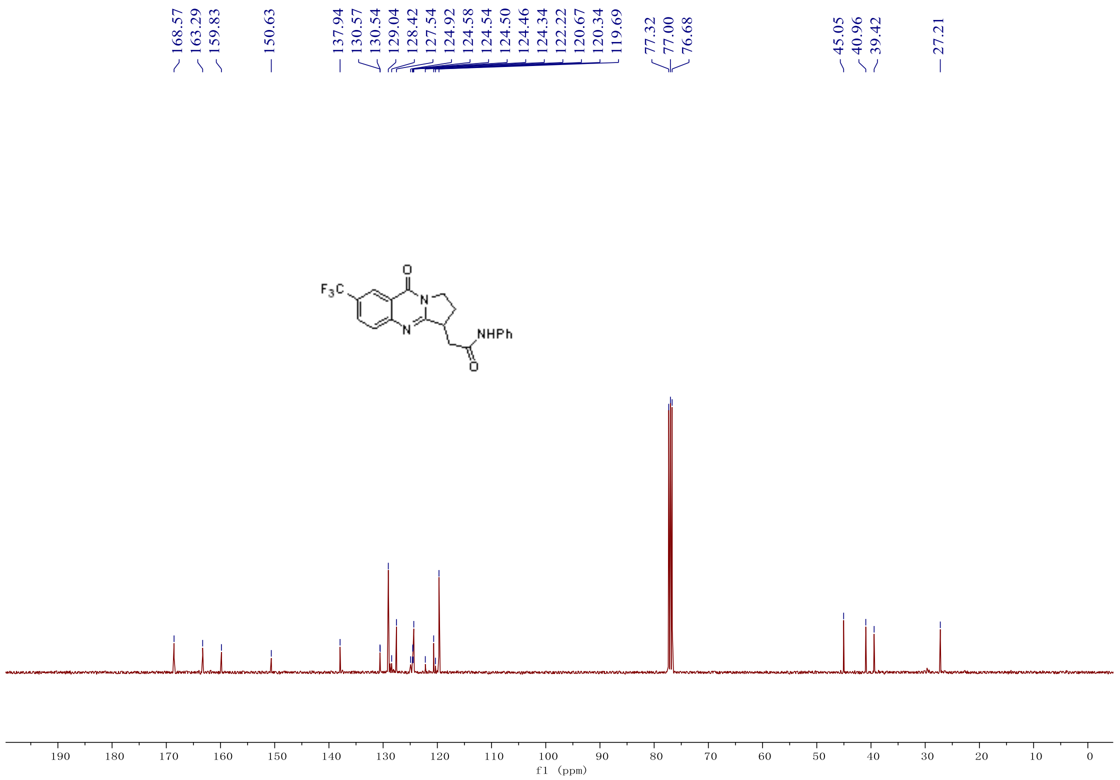

Figure S40 ^{13}C NMR spectra of **3ea**

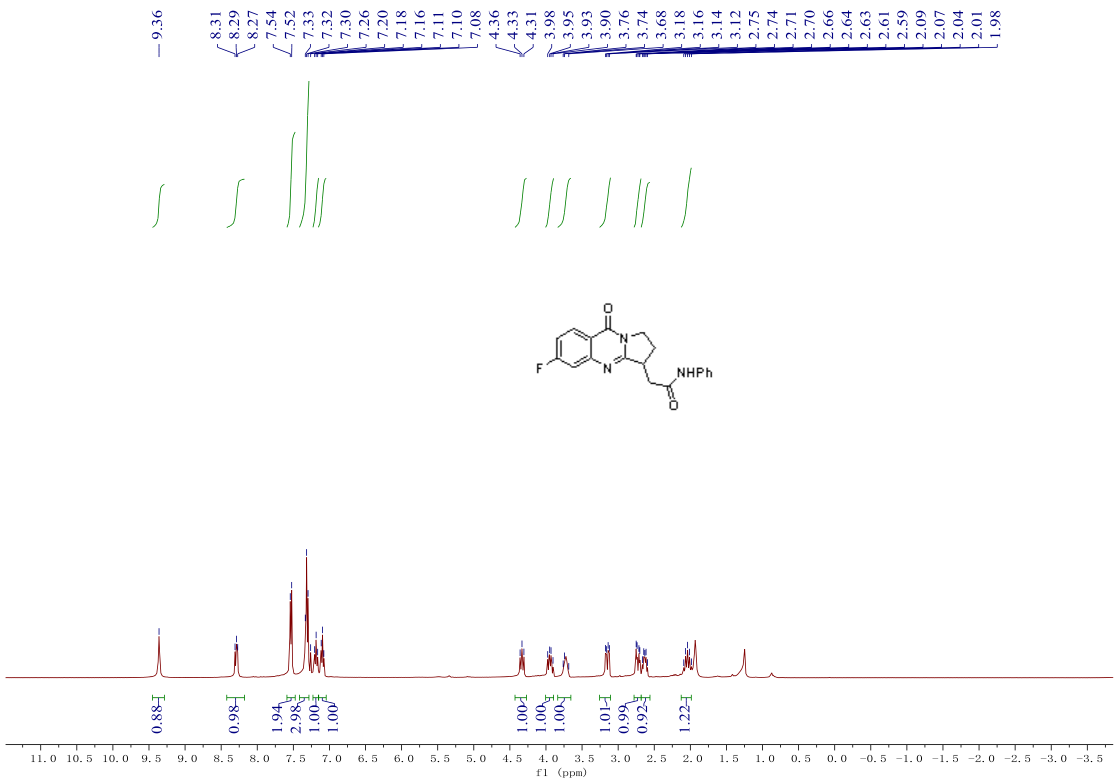

Figure S41 ^{1}H NMR spectra of **3fa**

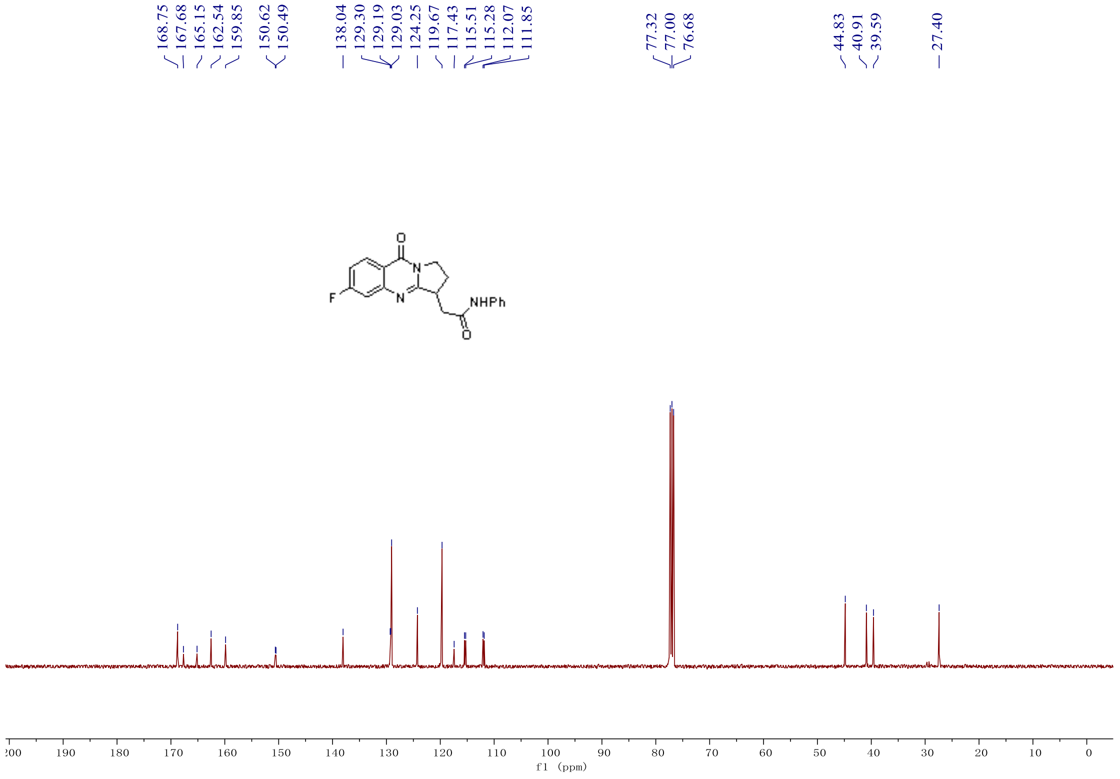

Figure S42 ^{13}C NMR spectra of **3fa**

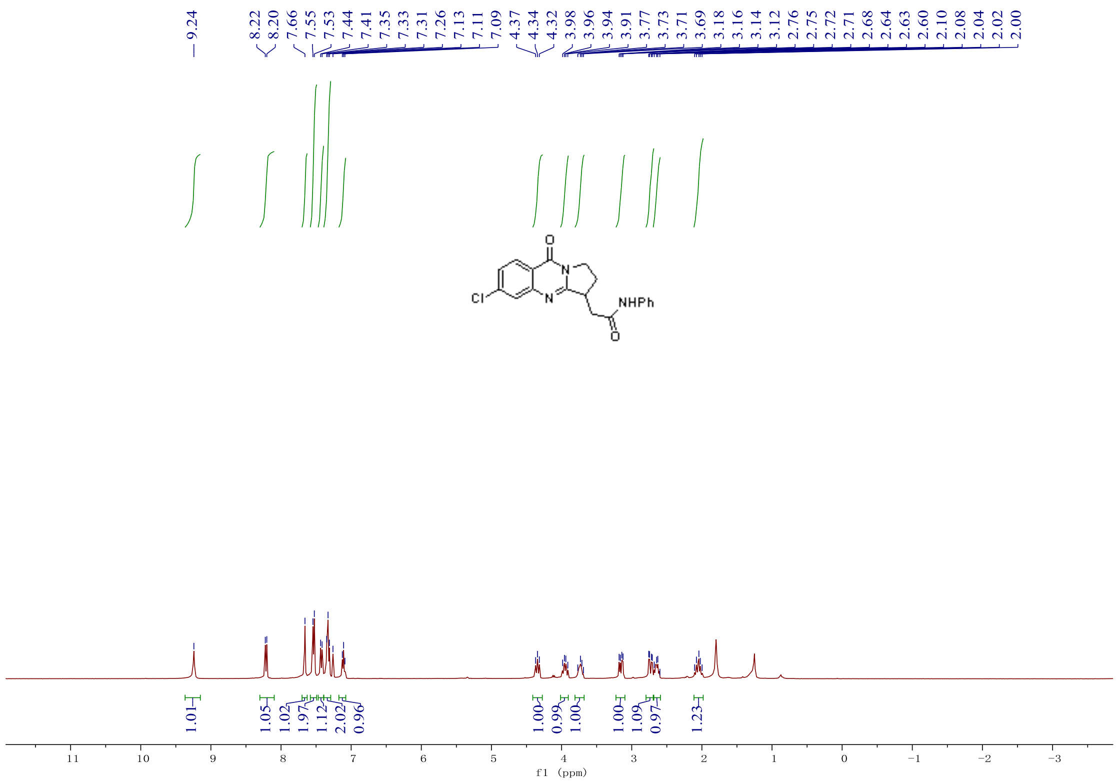

Figure S43 ^{1}H NMR spectra of **3ga**

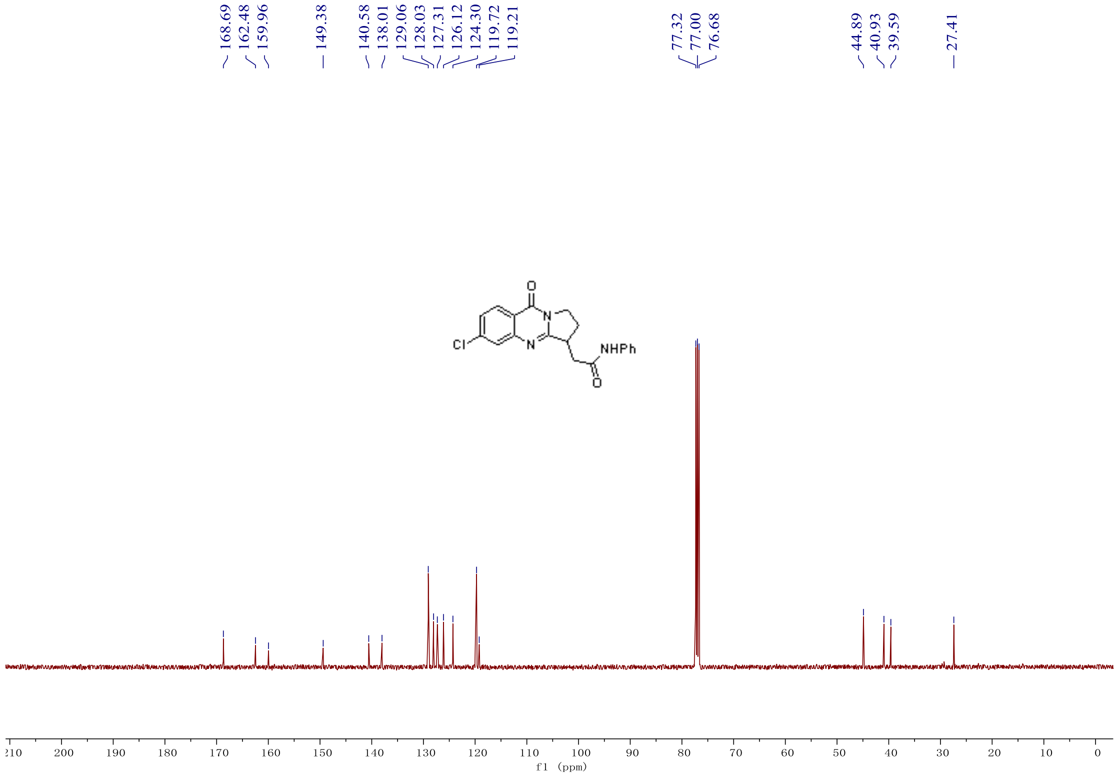

Figure S44 ^{13}C NMR spectra of **3ga**

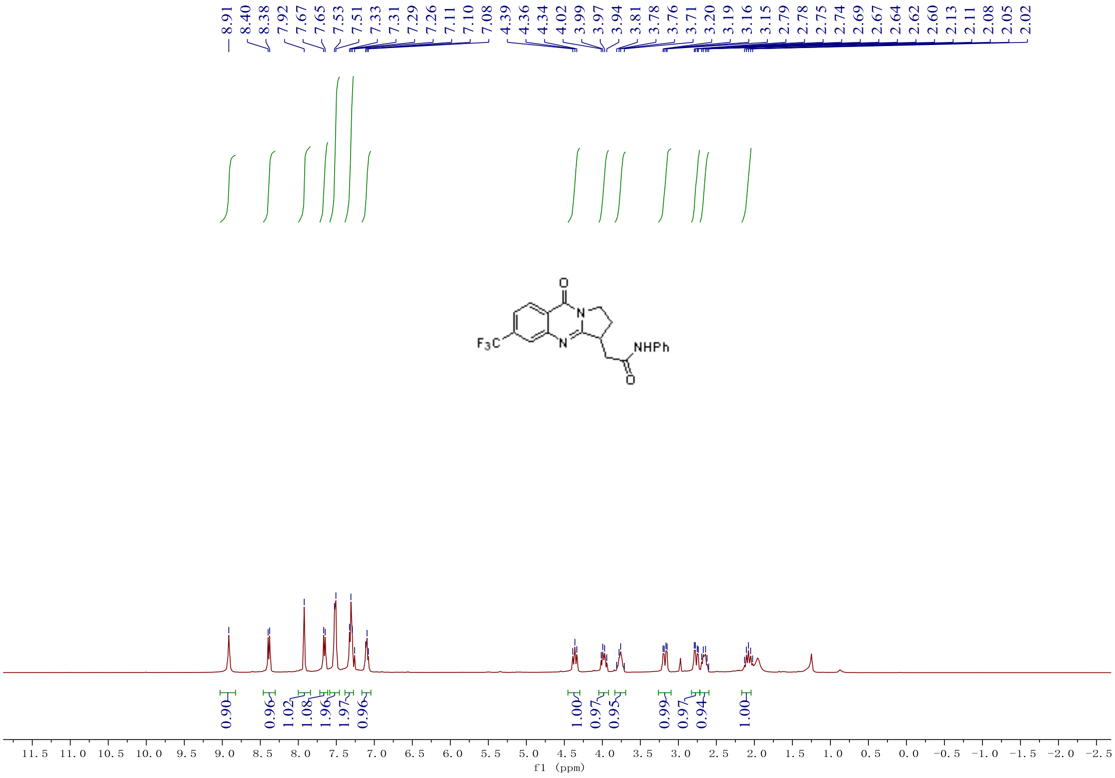

Figure S45 ^{1}H NMR spectra of **3ha**

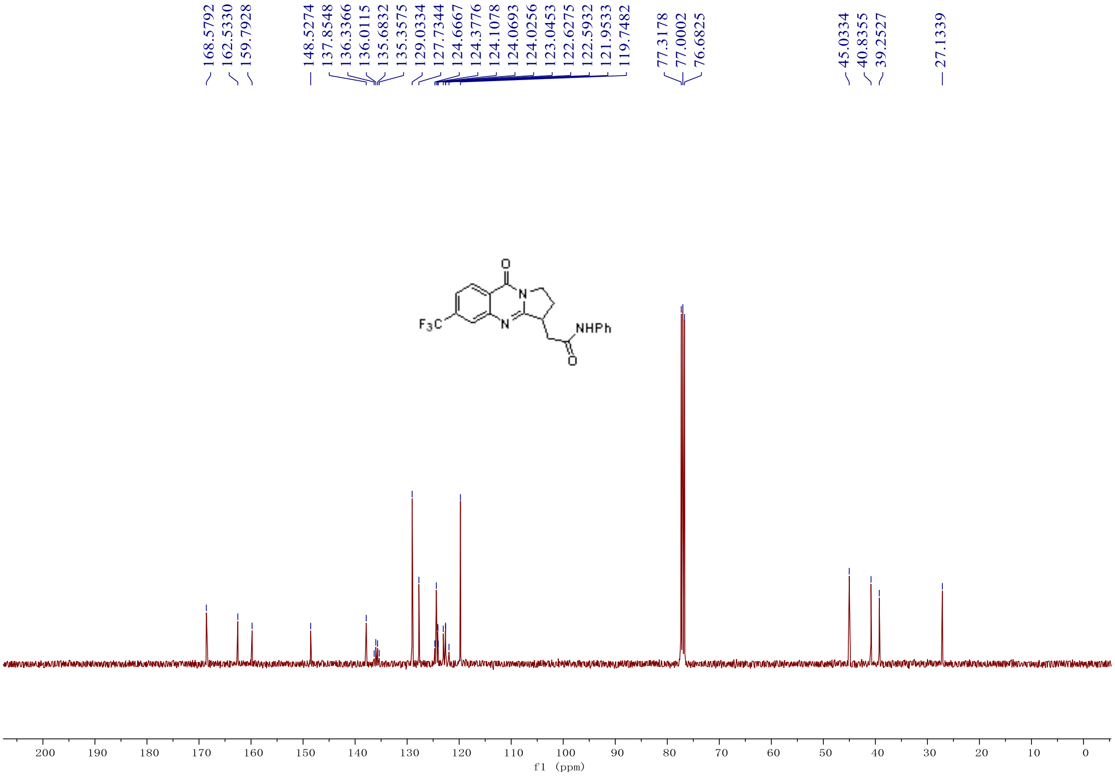

Figure S46 ^{13}C NMR spectra of **3ha**

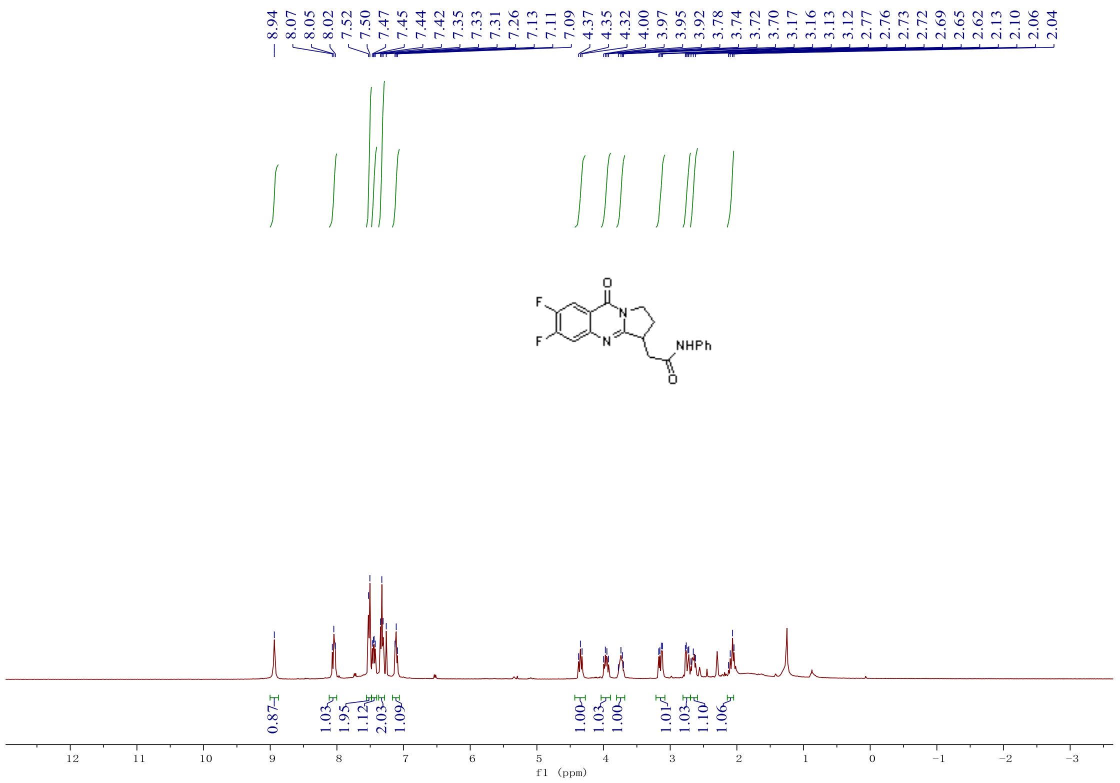

Figure S47 ^{1}H NMR spectra of **3ia**

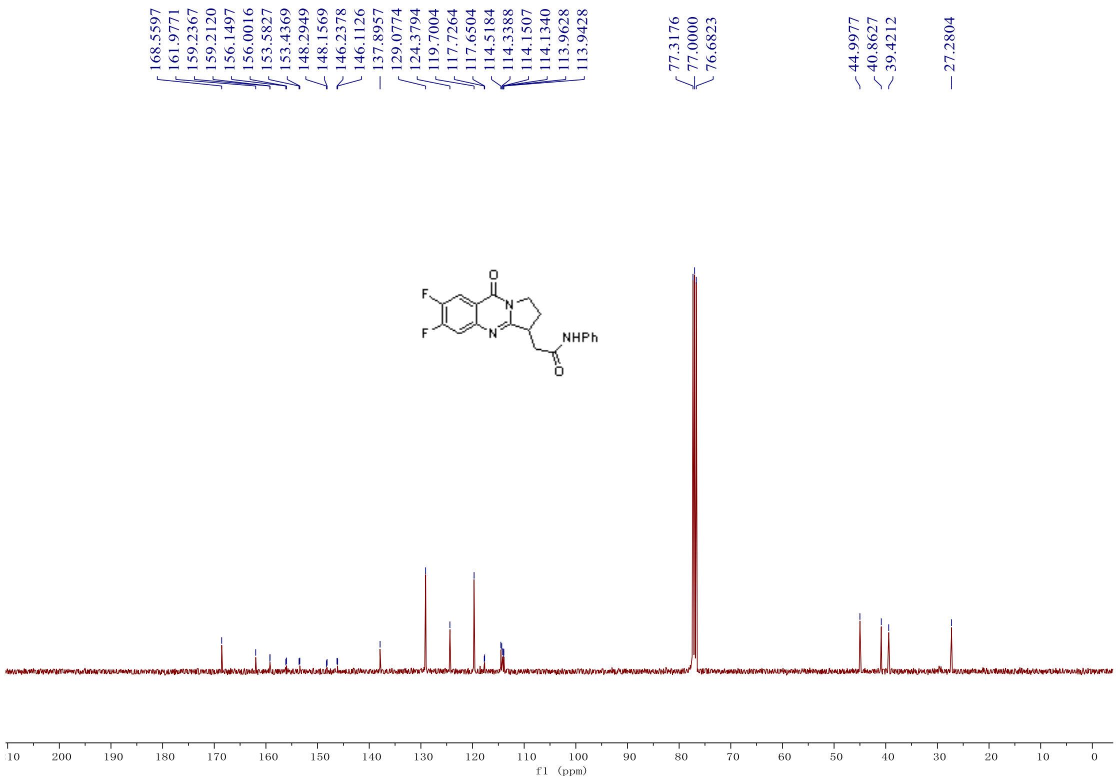

Figure S48 ^{13}C NMR spectra of **3ia**

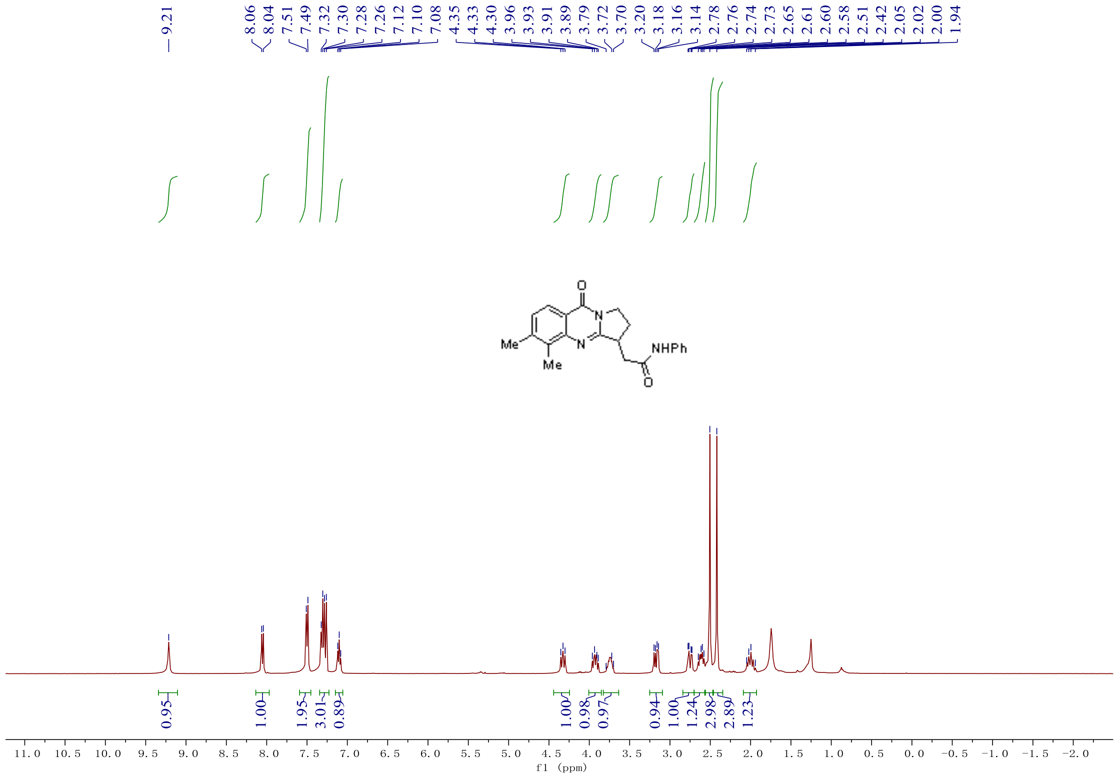

Figure S49 ¹H NMR spectra of **3ja**

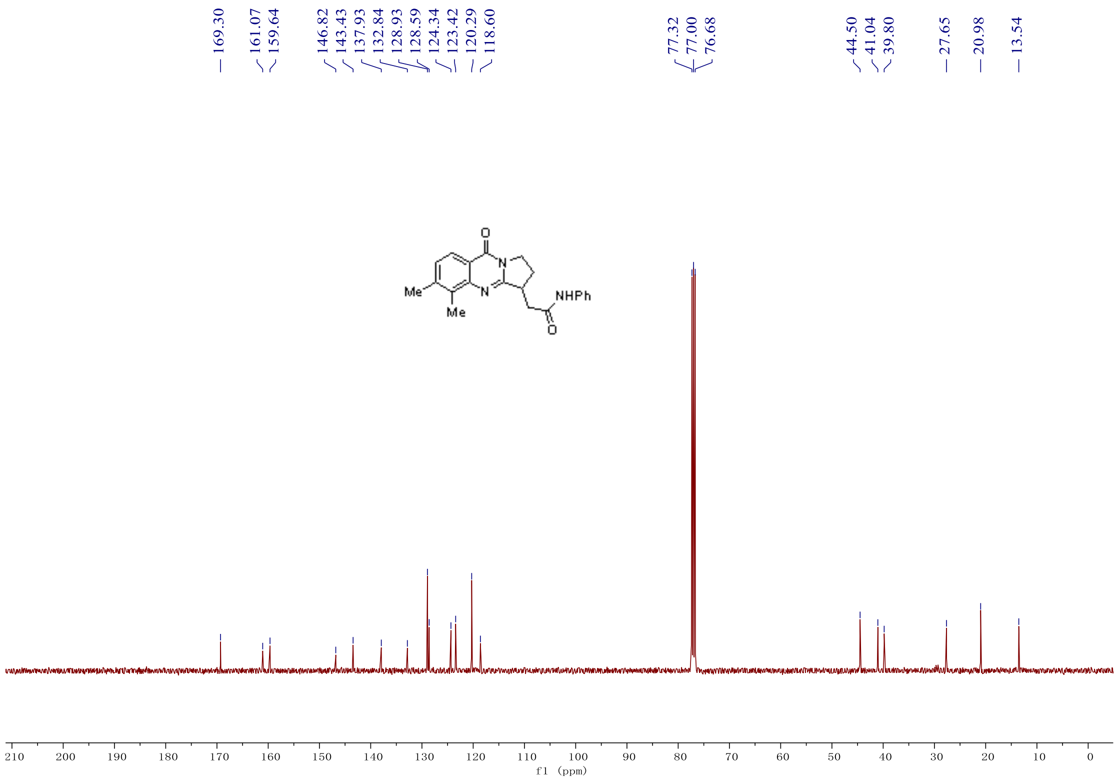

Figure S50 ¹³C NMR spectra of **3ja**

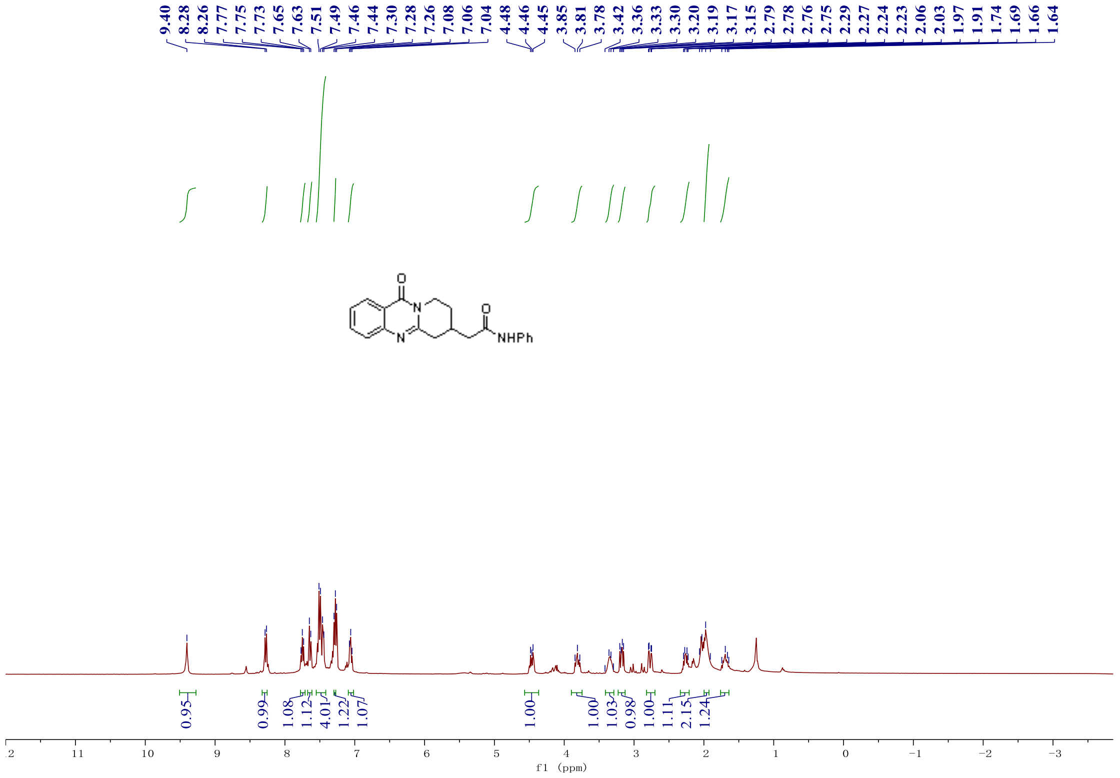

Figure S51 ^{1}H NMR spectra of **3ka**

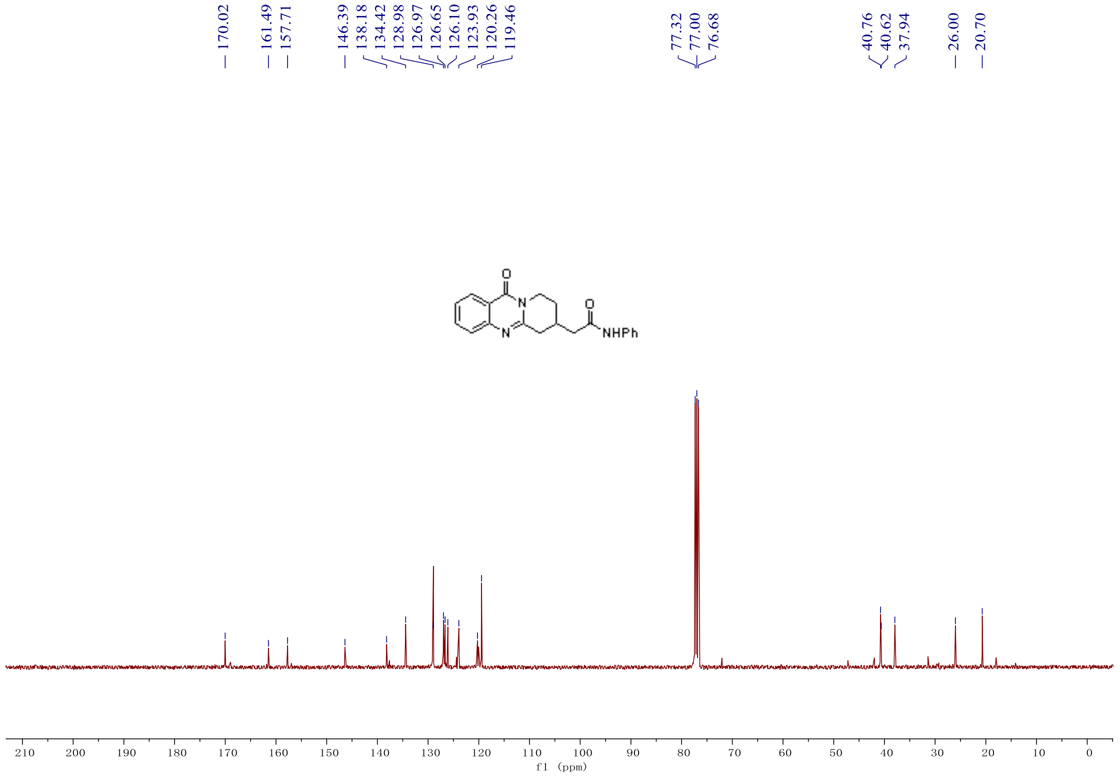

Figure S52 ^{13}C NMR spectra of **3ka**
